# Protein Sizing with 15 nm Conical Biological Nanopore YaxAB

**DOI:** 10.1021/acsnano.3c02847

**Published:** 2023-07-17

**Authors:** Sabine Straathof, Giovanni Di Muccio, Maaruthy Yelleswarapu, Melissa Alzate Banguero, Carsten Wloka, Nieck Jordy van der Heide, Mauro Chinappi, Giovanni Maglia

**Affiliations:** † Groningen Biomolecular Sciences & Biotechnology Institute, University of Groningen, 9747 AG Groningen, The Netherlands; ‡ Department of Industrial Engineering, University of Rome Tor Vergata, Via del Politecnico 1, 00133 Rome, Italy; ∥ Experimental Ophthalmology, Department of Ophthalmology, Charité - Universitätsmedizin Berlin, A Corporate Member of Freie Universität, Humboldt-University, The Berlin Institute of Health, Berlin 10178, Germany

**Keywords:** nanopores, electrophysiology, folded protein analysis, single-molecule, electroosmosis

## Abstract

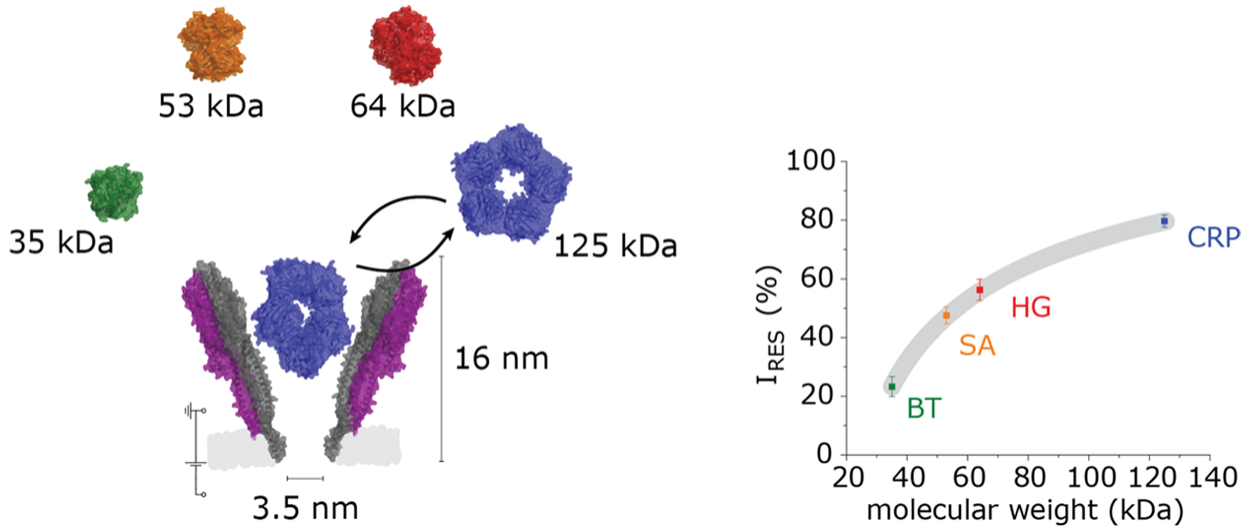

Nanopores are promising single-molecule tools for the electrical identification and sequencing of biomolecules. However, the characterization of proteins, especially in real-time and in complex biological samples, is complicated by the sheer variety of sizes and shapes in the proteome. Here, we introduce a large biological nanopore, YaxAB for folded protein analysis. The 15 nm *cis*-opening and a 3.5 nm *trans*-constriction describe a conical shape that allows the characterization of a wide range of proteins. Molecular dynamics showed proteins are captured by the electroosmotic flow, and the overall resistance is largely dominated by the narrow *trans* constriction region of the nanopore. Conveniently, proteins in the 35–125 kDa range remain trapped within the conical lumen of the nanopore for a time that can be tuned by the external bias. Contrary to cylindrical nanopores, in YaxAB, the current blockade decreases with the size of the trapped protein, as smaller proteins penetrate deeper into the constriction region than larger proteins do. These characteristics are especially useful for characterizing large proteins, as shown for pentameric C-reactive protein (125 kDa), a widely used health indicator, which showed a signal that could be identified in the background of other serum proteins.

## Introduction

Nanopores are nanometer-sized water-filled conduits in insulating membranes. Under a transmembrane applied potential, molecules entering the nanopore are characterized by specific changes of the ionic current.^1−6^ After their successful application in DNA sequencing, nanopores are now sought after for the characterization of proteins.^6^ Proteins, however, show a high level of heterogeneity in charge and shape, can be post-translationally modified, and are often present in few copies, for which identification with single-molecule resolution is most-likely required.^3,4,6^ At present, few single-molecule techniques exist that can characterize proteins. Mass spectrometry (MS) has single-molecule resolution, but currently it can only investigate large proteins or complexes.^7−9^ Fluorescence spectroscopy can address single molecules. However, fluorescent labels must be introduced through covalent or reversible interactions, which limits the identifications of a few specific features in proteins.^3^ Other techniques such as nanoelectromechanical systems have also been investigated, but at the moment they lack molecular resolution.^10^

Nanopores offer single-molecule identification of unlabeled molecules. An important characteristic of nanopores is that they can be integrated into low-cost and portable electronic devices for next-generation medical diagnostics. In the medical field, such devices would allow fast and efficient detection of biomarkers related to infections and diseases as well as progression and recovery of patients undergoing treatment. At present, many biomarkers can be identified using MS or enzyme-linked immunosorbent assay (ELISA). However, these techniques do not allow continuous detection, which is often needed, for example, in intensive care units.

Several nanopore-based approaches could be used to identify proteins. In a method borrowed from DNA sequencing, peptides or proteins are addressed as they are enzymatically translocated across a nanopore.^11^ In another approach reminiscent of MS analysis, specific proteases are used to cut proteins into peptides, which are then identified using a nanopore.^12,13^ The incorporation of the peptidase directly above the nanopore would allow single-molecule resolution.^14^ Real-time and continuous identification of proteins might also be obtained, as intact proteins are captured by a nanopore. Large modifications such as ubiquitination have been studied using this approach,^15,16^ and single point mutations in folded proteins have been detected.^17^ In fact, when a protein fits very precisely within the lumen of the nanopore, ionic current can detect tiny changes such as proteins’ conformational changes.^18−20^

One of the main challenges using solid-state nanopores for protein detection is that freely translocating molecules diffuse too quickly to be sampled by ionic currents.^21−24^ Trapping proteins in solid-state nanopores was recently shown with DNA origami functionalization,^25^ but this approach remains to be optimized for the detection of conformational changes in proteins with high resolution. To maximize molecular recognition, the size of the nanopore should be similar to the size of the protein analyte.^23,26^ As proteins exist in a variety of sizes and shapes, nanopores with different diameters should be used to sample proteins with different size. At present, only a few nanopores exist that can sample folded proteins. MspA^16,27,28^ and FraC^29^ have been used to sample small proteins (5–25 kDa), while ClyA^18−20,30−32^ and PlyAB^17,33^ have been shown to sample proteins in the 20–80 kDa mass range.

**Figure 1:**
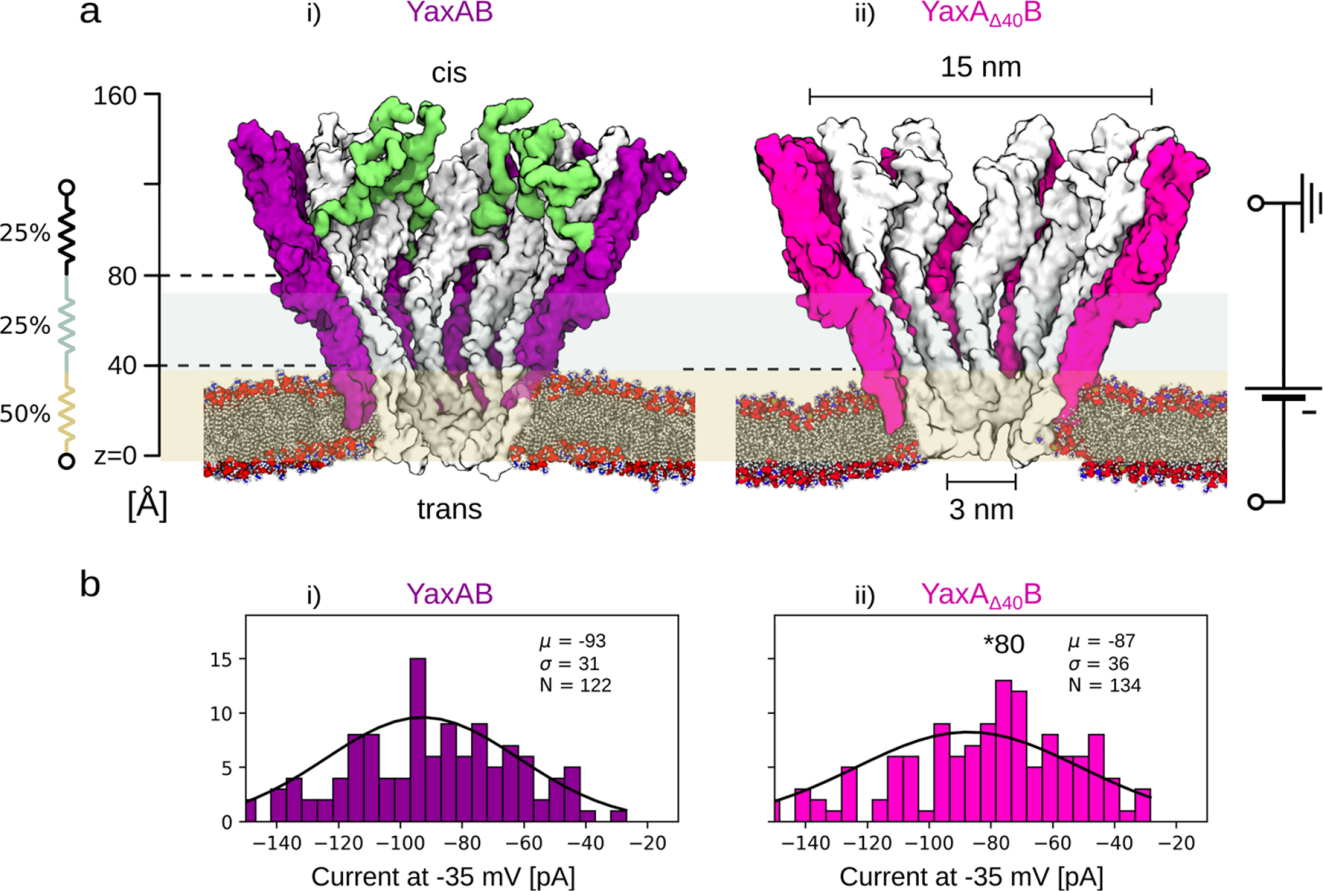
YaxAB nanopore characterization. (a) Molecular surface illustration of a cut-through of the didecameric YaxAB (i) and YaxA_Δ40_B (ii). YaxA and YaxA_Δ40_ monomers are represented in purple and magenta, respectively; the N-terminal tail of YaxA is in green; YaxB monomers are in white. The molecular models were obtained using MODELLER,^36^ starting from the PDB structure 6EL1.^34^ (b) Experimental distribution of open pore currents, measured at −35 mV for the (i) YaxAB (*N* = 112 pores from 7 independent recordings) and (ii) modified YaxA_Δ40_B (*N* = 134 pores from 8 independent recordings). Measurements were performed in 150 mM NaCl and 15 mM TrisHCl pH 7.5. Data were recorded with a 50 kHz sampling rate and 10 kHz Bessel filter. The smeared distributions most likely reflect the fact that YaxAB assembles in different multimeric forms. Gaussian fits (mean μ and standard deviation σ) of the distributions are reported to guide the eye. The *80 indicates the most prevalent assembly for the YaxA_Δ40_B pores. Pictures in panel (a) are realized with VMD^37^ software. Deleted sequence in YaxA_Δ40_B: TQTQLAIDNVLASAESTIQLNELPKVVLDFITGEQTSVAR; see Figure S2.

Here, we detect folded proteins with α-helical pore-forming toxin (PFT) from *Yersinia enterocolitica*, YaxAB,^34^ which is composed of multiple subunits of YaxA and YaxB dimers. Electron microscopy revealed that the pore can assemble with a range of sizes, ranging from 8 to 12 dimeric subunits, with the didecamer being the most represented form.^34^ Didecameric YaxAB has a conical shape with 15/3.5 nm *cis*/*trans* openings (Figure 1), making the YaxAB characterized here the largest biological nanopore to date for folded protein analysis (Figure S1). The nanopore shape allowed the trapping of a variety of proteins (35–125 kDa). Intriguingly, the residual current signal was inversely proportional to the size of the protein, which is opposite to what was observed in cylindrical pores (e.g., ClyA^30,31^ and PlyAB^33^). YaxAB appeared especially valuable for the characterization of large proteins such as C-reactive protein (CRP), a 125 kDa biomarker of which the blood concentration spikes to up to 350 mg/L during inflammation.^35^ CRP showed a unique current level, which was not observed with other proteins in depleted serum. Hence, YaxAB might find application in protein sizing using nanopores or as a real-time sensor for inflammation in continuum devices for clinical diagnostics.

## Results and Discussion

### Characterization of YaxAB Nanopores

Structural analysis revealed that YaxAB forms a conical transmembrane nanopore (PDB: 6EL1) composed of YaxA and YaxB heterodimers, where YaxA occupies the exterior, and YaxB the interior and transmembrane region of the nanopore rings (Figures 1a and S2).^34^ CryoEM analysis also showed that the nanopore can assemble in various stoichiometries, ranging from 8 to 12 heterodimers, with the didecamer pore most frequently observed.^34^ The first 40 amino acids (aa) of the monomer YaxA are not resolved in the crystal structure,^34^ suggesting they form flexible random coils. The 40 aa N-terminal tail is composed of five acidic and two basic residues; hence, the total charge at pH 7.5 is about −3e per chain and a total of −30e for didecameric YaxAB. We prepared two variants of YaxA, the unmodified (YaxA) and a truncated version, where the first 40 aa were deleted (YaxA_Δ40_; Figures 1 and S2). Each YaxA variant was then oligomerized with the YaxB subunits. Both unmodified YaxAB and the YaxA_Δ40_B variant assembled into lipid membranes forming conductive nanopores. Both nanopore families showed a multiple conductance distribution (Figure 1b), suggesting that, as observed in CryoEM, also on lipid membranes, YaxAB assembles with different nanopore stoichiometries. When applying −35 mV, the currents showed a broad distribution centered at −93 ± 31 pA for YaxAB and −87 ± 36 pA for YaxA_Δ40_B nanopores. We continued with the most prevalent YaxAB and YaxA_Δ40_B corresponding to pores with a conductance of −80 pA ± 8 pA at −35 mV in 150 mM NaCl and 15 mM TrisHCl pH 7.5. These pores, which in the following will be referred to as YaxAB^80^ and YaxA_Δ40_B^80^ respectively, likely correspond to the didecameric nanopores.

### Ion Transport across YaxAB Nanopores

**Figure 2:**
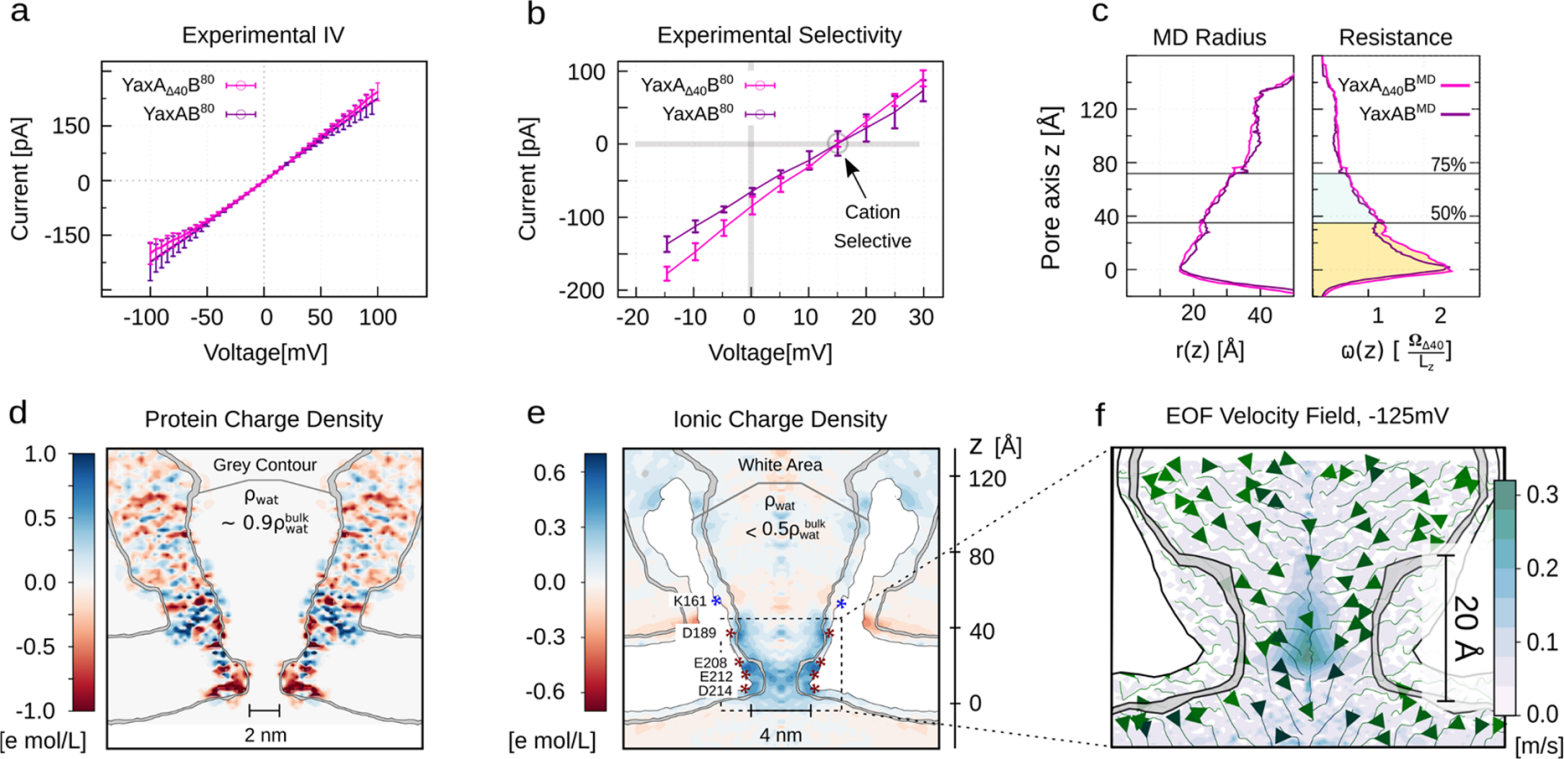
Ionic transport across YaxAB nanopores. (a) *I*/*V* curves of full-length YaxAB^80^ and truncated YaxA_Δ40_B^80^. (b) Reverse potential of YaxAB^80^ and YaxA_Δ40_B^80^, experimentally measured with 300 (*cis*) and 75 mM NaCl (*trans*) chamber concentrations. (c) Effective radius of the inner electrolyte volume and related resistance-per-unit-length ω(z), along the pore axis *z*. ω(*z*) is normalized by the average resistance-per-unit-length of the YaxA_Δ40_B system, , with Ω_Δ40_ being the total resistance and *L*_*z*_ = 15 nm length of the pore. (d,e) Radial average of the charge density computed by equilibrium MD (d) over the didecameric YaxA_Δ40_B atoms and (e) over the ions in the electrolyte, 0.15 M NaCl. (f) Electroosmotic velocity field, computed by nonequilibrium MD simulations at Δ*V* = −125 mV for the didecameric YaxA_Δ40_B nanopore. The gray contours in panels (d)–(f) represent the area where the water density (ρ_wat_) is below 0.9 of the water bulk density (ρ^bulk^_wat_). The white area in panels (e) and (f) represents the region where the water density goes below 0.5 ρ^bulk^_wat.·_ All the nonequilibrium MD simulations are sampled for 130 ns, saving the coordinates every 20 ps.

We found that, despite the presence of the N-terminal tails in the full-length YaxAB, the two pores exhibited similar *I*/*V* curves (Figure 2a) and ion selectivities (Figure 2b). Both YaxAB^80^ and YaxA_Δ40_B^80^ are cation selective (*P*_Na+_/*P*_Cl–_ = 2.60 ± 0.09 and 2.46 ± 0.22, respectively, as calculated from the pores’ reverse potential under asymmetric salt conditions and the Goldman–Hodgkin–Katz equation, eq 1, see Methods). These observations suggest that the YaxA tails do not significantly influence the passage of ions through the nanopore. Nanopores with higher conductance showed *I*/*V* curves with similar shapes (Figure S3), suggesting that the shape of the different nanopores is similar despite their oligomeric composition.

To characterize the nanofluidic properties of YaxAB nanopores further, we performed MD simulations of the didecameric version of the YaxAB and YaxA_Δ40_B nanopores. Quasi-1D approximation^38,39^ of the didecameric YaxAB and the YaxA_Δ40_B revealed that about 50% of the total resistance of the nanopore is in the first 3.5 nm from the trans entry of the nanopore, which we refer to as the constriction region (Figure 1a yellow, Figure 2c, Figure S4). The YaxA tails fluctuate at the top of the nanopores, where they contribute little to the total resistance, which explains the similar ion selectivity of YaxAB and YaxA_Δ40_B that was experimentally observed. By using the average electrolyte conductivity of NaCl in 0.15 M (1.9 S/m),^40^ the estimated conductance of the YaxA_Δ40_B and YaxAB nanopores conductivity was 2.55 ± 0.2 and 2.85 ± 0.2 nS, respectively, corresponding to 90 ± 10 and 100 ± 10 pA at +35 mV. Hence, both pores exhibit similar conductance. This result is also confirmed by nonequilibrium MD simulations: at ±125 mV, the currents of the didecameric YaxA_Δ40_B and YaxAB are indistinguishable, in agreement with the experimental observations (Figure S4).

MD simulations (Figure 2c) also showed that the nanopore constriction region (0 < *z* < 35 Å) is predominantly negatively charged (Figure 2d), which promotes the accumulation of cations (Figure 2e). Under an applied bias, the electric field exerts a net volume force on the charged regions of the electrolyte, generating an EOF in the direction of the cation flow (Figure 2f). The total electrical force acting on the electrolyte solution (i.e., the Coulombian force pushing the free moving ions) and the electroosmotic flow (EOF) at ΔV = ± 125 mV was calculated by nonequilibrium MD simulations (Figure S4), resulting in a net force for the YaxA_Δ40_B nanopore of −119 ± 92 pN at −125 mV, corresponding to an EOF of −61.3 ± 3 molecules/ns. YaxAB showed slightly higher values than YaxA_Δ40_B, which can probably be ascribed to the negatively charged unstructured N-tails, which attract additional cations inside the pore. The maximum water velocity field inside the constriction region for the YaxA_Δ40_B pore (Figure 2f) is about 0.3 m/s, which is larger with respect to other commonly employed smaller nanopores, such as ClyA (<0.21 m/s^41^ at 100 mV, bicylindrical nanopore, 5.5–3.5 nm *cis*-*trans* diameters), FraC (<0.23 m/s^29^ at 100 mV, conical nanopore, 5.5 nm *cis*-diameter 1.5 nm *trans*-diameter, extrapolated values) and the α-Hemolysin (0.06 m/s^39^ at 125 mV for pH 7, 0.1 m/s^42^ at 125 mV at pH 2.8, see also refs.,^43,44^ cylindrical transmembrane region with ∼2 nm^43^ diameter). A similar average EOF velocity was also reported for artificial DNA-origami nanopores (0.3 m/s^45^ at 100 mV for a cylindrical nanopore of ∼2 nm diameter).

In YaxAB, three negatively charged rings (E208, E212 and D214) facing toward the pore lumen and lying in the constriction region (see Figures 2e and S5 for details). We expected that these residues might govern the ionic selectivity of the nanopore and therefore the EOF. MD results showed that by neutralizing the charge of the three acidic rings (i.e., mutating them into neutral asparagines, E208N E212N D214N, NNN system) the EOF reduced to zero (Figure S6). By substituting the three rings into positively charged arginines (E208R E212R D214R, RRR system), the EOF was reversed with respect to the YaxA_Δ40_B. In agreement with the MD simulations, we observed an inverse of ion selectivity: 0.80 ± 0.04 and 0.72 ± 0.01 for the NNN and the RRR system, respectively (Figure S7). It should be noted that, in the experimental setup, the NNN system presents a lower current distribution with respect to the YaxA_Δ40_B^80^, possibly corresponding to a smaller multimer assembly (Figure S8). Despite the constriction being neutralized, the pore is slightly anion selective (Figure S7), in accordance with the MD data (Figure S6 panel a).

### Protein Capture and Discrimination with YaxAB Nanopores

**Figure 3:**
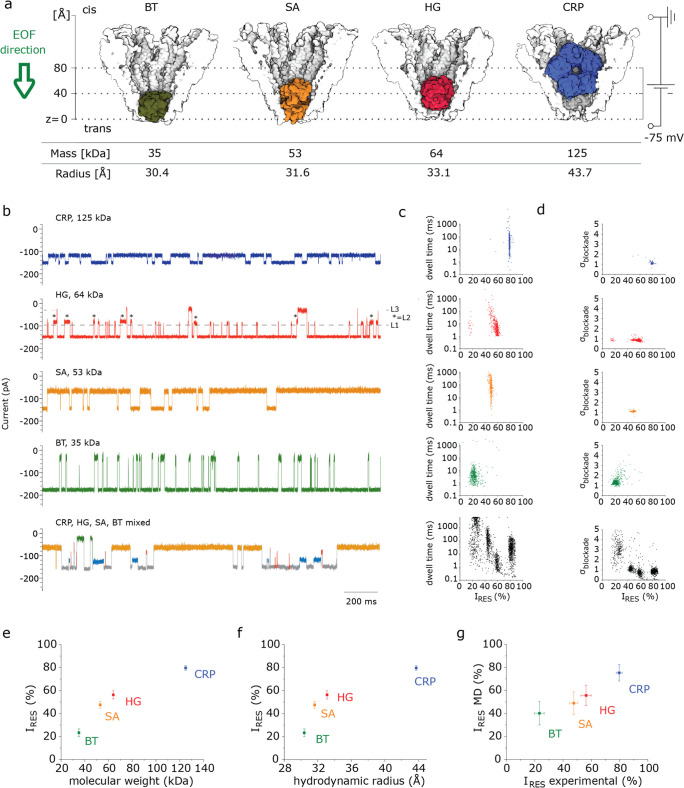
Characterization of blockades for four different proteins trapped in YaxA_Δ40_B^80^. (a) Sketch of the four proteins inside the nanopore. Pictures are taken from the last frame of the 35 ns SMD simulations. (b) Electrophysiology traces of individual proteins at 120 nM; and 50 nM BT, 50 nM CRP, 50 nM HG, and 20 nM SA mixed in *cis*. Proteins were captured at −75 mV applied potential. Data was filtered with additional 5 kHz low-pass Gaussian filter for visualization; the mixed trace was filtered with 2 kHz low-pass Gaussian filter for analysis. .(c) Dwell time vs *I*_RES_ and (d) σ_blockade_ vs *I*_RES_ scatterplot of events in panel (b). Individual proteins contain *n* = 600 data points each, the mixed trace contains *n* = 1694 data points. See also Figures S18 and S24e) The inverse relation of *I*_RES_ and molecular weight; (f) *I*_RES_ and hydrodynamic radius, i.e., larger protein CRP has larger *I*_RES_. (g) Comparison of single protein experimental *I*_RES_ with those computed by molecular dynamics (*I*_RES_ MD). Resistance profiles from MD are reported in Figure S22. Error bars correspond to the standard deviation of each measure from at least three independent measurements.

Biological nanopores with a large diameter can be used to detect and study folded proteins.^30,33^ The ability of the YaxAB nanopore to capture proteins was tested using proteins of different sizes (Figure 3a): C-reactive protein (CRP, 125 kDa), haemoglobin (HG, 64 kDa), streptavidin (SA, 53 kDa), and bovine thrombin (BT, 35 kDa; Figure S9, Table 1, Table S1). Protein blockades were characterized by the residual current (*I*_RES_ = *I*_B_/*I*_O_ × 100%, where *I*_B_ is the current of the blockade and *I*_O_ is the open pore current), the duration of the event (dwell time, ms), and the normalized blockade noise (σ_blockade_; Figure S10). Protein blockades depended on a variety of factors, including the size of the nanopore (estimated from its conductance, Figure S11) and the applied potential used (see below). Therefore, we compared protein blockades to YaxA_Δ40_B^80^ with a conductance of 80 ± 8 pA.

**Table 1:** Protein Capture by YaxA_Δ40_B^80^^a^

protein	mass (kDa)	*r*_h_ (Å)	*I*_RES_ ± σ (%)	σ_blockade_ ± σ	*k*_on_ (μM^–1^ s^–1^)	*k*_off_ (s^–1^)
CRP^49^ in buffer	125 (pentamer)	43.74	79.7 ± 2.1	1.4 ± 0.2	308.9 ± 9.7	27.0 ± 0.7
CRP + serum			78.9 ± 0.5	1.2 ± 0.1	ND^b^	36.8 ± 6.1
HG^50^	64 (tetramer)	33.10	56.3 ± 3.6	0.95 ± 0.15	208.6 ± 8.9	189.1 ± 3.9
SA^51^	53 (tetramer)	31.62	47.6 ± 2.9	1.1 ± 0.2	473.2 ± 16.6	13.3 ± 0.3
BT^52^	35 (dimer)	30.38	23.3 ± 3.4	1.3 ± 0.1	150.9 ± 4.4	121 ± 8.7

a Mass and hydrodynamic radius (*r*_h_) were computed with HullRad^48^ software. The error in *I*_RES_ and σ_blockade_ represents the standard deviation (σ) of cumulated data points (*n* = 1800) from three technical replicates (*N* = 3 pores). The σ_blockade_ is normalized for the noise in the *I*_O_. The *k*_on_ and *k*_off_ are computed from the capture and release rates shown in Figure S23. Experiments were performed in 150 mM NaCl, 15 mM TrisHCl (pH, 7.5), with 120 nM protein in *cis*. Data was recorded with 50 kHz sampling rate and 10 kHz low-pass Bessel filter.

b Note that serum proteins also are captured by the nanopore and interfere with the measurement of *k*_on_.

Protein blockades were only observed when each of the four proteins was added to the *cis* side of the nanopore and when a negative potential was applied, indicating that, as expected, the proteins entered the nanopore from *cis* to *trans* following the EOF. When using YaxAB^80^ (Figure S12) and YaxA_Δ40_B^80^ nanopores (Figure S13), protein blockades were very similar, although the baseline of YaxAB^80^ would sometimes shift (Figure S12). This presumably reflects the flexible YaxA tails sometimes entering the sensing region, despite being repelled on account of their slight charge (−3e, Figure S2) by the electrostatic potential. We continued with YaxA_Δ40_B^80^ as it depicted a stable open pore current. In YaxA_Δ40_B^80^, the voltage dependency of protein blockades revealed that upon increasing the potential, CRP (Figure S14), HG (Figure S15), and SA (Figure S16) showed longer dwell times, suggesting these proteins did not translocate the nanopore. By contrast, BT showed shorter dwell times as the voltage increased (> –50 mV; Figure S17), suggesting that the protein translocated through the nanopore. In addition, we observed that small variations in the nanopore conductance, most likely corresponding to variations of the *trans* diameter, changed dramatically the dwell time of BT blockades (Figure S18). In turn, BT (hydrodynamic radius 30.4 Å, 35 kDa) provides a good indication of the smallest protein that can be trapped inside YaxA_Δ40_B^80^. The *I*_RES_ also decreased with the external bias (i.e., more current was blocked, Figures S19 and S20). This suggests that proteins were trapped deeper in the conical shape of the nanopore upon increasing EOF. Increasing the external bias augmented the capture frequency, which is also consistent with an EOF-driven capture^46^ (Figure S19).

At −75 mV, CRP, BT, and SA showed well-defined dwell time–*I*_RES_ clusters, whereas HG presented a more complex *I*_RES_ distribution (Figure 3c). Careful analysis of the blockades of the latter revealed three main populations: L1, L2, and L3, where L3 was often populating a deeper state (Figure S21). This behavior could rise from multiple factors. A likely explanation for L1 and L3 blockades is that HG binds with two separate trapping sites within the nanopore, a behavior observed before for HG in PlyAB^17^ and human thrombin in ClyA^30^ nanopores. The small differences observed by L1 and L2 blockades may originate from different orientations of the protein inside the nanopore (Figure S22), as also observed for substrate-binding protein in ClyA.^18,47^

The proteins showed similar capture frequencies (Figure S23), but their dwell time differed (Table 1). Notably, HG had a release frequency (τ^–1^ of dwell time; see Methods and Figure S10) about one order of magnitude faster than those of the other trapped proteins (SA and CRP, Figure S23, Table 1). Similar to BT, HG dwell time was shorter in pores with slightly larger conductance (Figure S18). This possibly reflects unfavorable steric interactions between the HG and YaxAB and requires further investigation. Overall, YaxA_Δ40_B^80^ was able to differentiate CRP, SA, HG, and BT when all proteins were added simultaneously to *cis* (Figure 3b, c) and the resolution of cluster separation could be finetuned (Figure S24).

Interestingly, although for nonspherical proteins (such as CRP) and noncylindrical pores, the relationship between the hydrodynamic radius and the current blockade might be complex, we observed a good correlation between the average *I*_RES_ and the dimensions (molecular weight and hydrodynamic radius, Table 1) of the four different proteins. The *I*_RES_ of each protein at a given voltage increases with the protein dimensions, with the larger molecule CRP showing the highest *I*_RES_, and the smaller BT showing the lowest *I*_RES_ (Figure 3e,f and Table 1). This behavior is in apparent contrast with the nanopore resistive pulse-behavior in cylindrical nanopores such as ClyA^30,31^ and PlyAB,^33^ where larger proteins tend to give a larger current blockade (i.e., low *I*_RES_). This observation can be explained considering that 50% of the electrical resistance is focused into the pore constriction region (Figure 2). It would be likely that smaller proteins can penetrate the nanopore deeper, hindering the highly resistive part of the nanopore more. Molecules with larger volume would not effectively occupy the constriction and would allow for more residual current. This hypothesis was tested by Steered-MD (SMD) simulations, where each protein was pulled by a constant force from the *cis* to the *trans* side of the nanopore. As expected, the average steady state position *z̅* reached by each protein was inversely proportional to the volume of the protein, with the CRP protein dwelling at the center of the pore (*z̅*_CRP_ ∼ 80 Å) and the BT dwelling deep inside the constriction (*z̅*_BT_ < 20 Å; Figure 3a). Translocation events across the pore constriction were not observed within the simulation time. The theoretically computed *I*_RES_ obtained from the last frames of the simulations were in reasonably good agreement with respect to the experimental ones (Figure 3g, Figure S22). For the CRP case, an additional contribution to the total current (∼10%) would also come from lateral fenestrations between the YaxAB helices; see Pore Resistance Methods.

### Discrimination of CRP in the Presence of Depleted Serum

**Figure 4:**
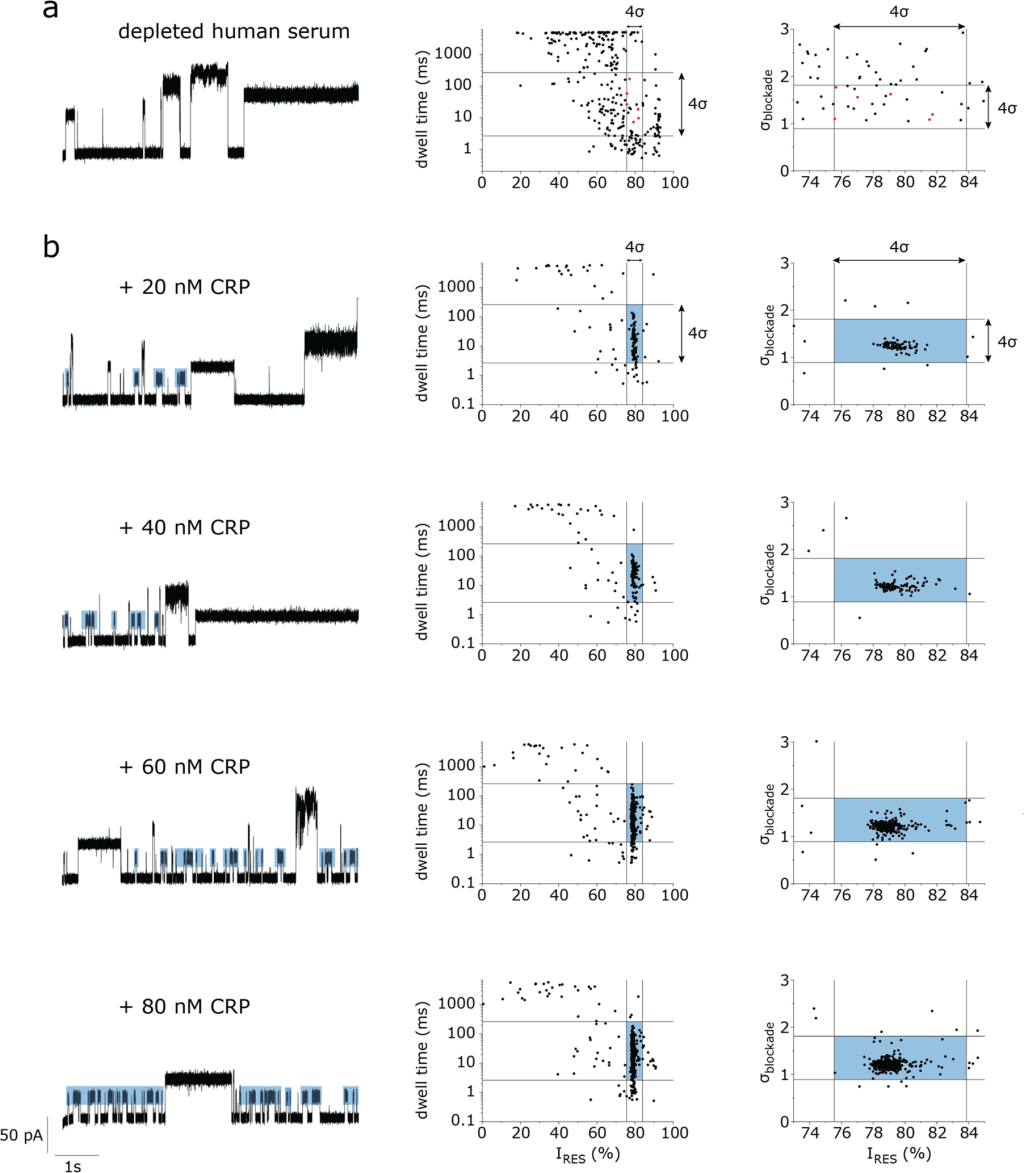
Discriminating CRP in the presence of depleted human serum. (a) 10 min recording of depleted human serum proteins captured by YaxA_Δ40_B^80^. The trace shows representative blockades (left), the dwell time–*I*_RES_ scatterplot (middle) and the σ_blockade_–*I*_RES_ scatterplot (right). The red data points (6 out of 297) are in an 2σ-interval around the mean for *I*_RES_, σ_blockade_, and dwell time of CRP. (b) CRP blockades in the background of depleted human serum. Left, representative current trace is shown, with CRP blockades annotated in blue. Middle, dwell time–*I*_RES_ scatterplot. Right, σ_blockade_–*I*_RES_ scatterplot. The boundaries indicate the residues within 2σ of the mean values for CRP blockades in buffer (150 mM NaCl, 15 mM TrisHCl pH 7.5). Traces in (b) were recorded for 2 min. Experiments were performed in 150 mM NaCl and 15 mM TrisHCl at pH 7.5, with a 50 kHz sampling rate and 10 kHz low-pass Bessel filter. For analysis, data were filtered with an additional 5 kHz low-pass Gaussian filter.

In the clinic, CRP is routinely measured with point-of-care diagnostics to monitor patients at risk for cardiovascular events and autoimmune diseases, and it is an important parameter when prescribing antibiotics. The reference CRP concentration in human serum is <0.2–10.5 mg/L^35,53^ for healthy adults and can increase to 40–350 mg/L^35^ during inflammation, with typical values for viral infections (10–40 mg/L^53^), bacterial infection (>200 mg/L^53^), and cardiovascular risk (systemically >3 mg/L^54^), with 3 mg/L corresponding to 24 nM of CRP. We next tested whether YaxA_Δ40_B^80^ could also detect CRP in the presence of a biologically complex sample: human serum. We depleted human serum for the top 14 most abundant proteins, such as IgG, albumin, and transferrin (see Methods). Typically, high concentration of serum dramatically reduced the stability of the lipid bilayer. We found that adding 2.5 μL of depleted human serum to the *cis* side of the nanopore (160-fold dilution) allowed electrical recordings for a few minutes. Under an applied potential of −75 mV, we found that proteins from depleted serum induced blockades with an *I*_RES_ of 20–80% (Figures 4a and S25). Occasionally, serum proteins remain trapped inside the nanopore for several seconds. In these instances, the potential was reversed to release the protein. Among the blockades recorded in 627 s, just 6 events could be assigned to CRP (events within 2σ of the *I*_RES_, σ_blockade_ and dwell time; see Methods, Figure S26), which is consistent with the trace presence of CRP in serum. This indicates that most likely no other serum proteins induced a blockade similar to CRP. The addition of 20–80 nM (2.5–10 mg/L) of CRP in the background of 2.5 μL of depleted serum induced several blockades (Figure 4b), which were highly similar to CRP in buffer (Figures S26, S27; Table S2), further indicating that proteins in depleted serum do not alter the signature blockade of CRP. Therefore, if an amphipathic membrane can be made to withstand undiluted blood, this approach would be capable of measuring CRP directly from a biological sample.

## Conclusions

In recent years, biological nanopores have been investigated for the analysis, identification, and sequencing of single proteins. Small nanopores (diameter 1–2 nm) might be used to identify amino acids as they are linearly transported across the nanopore.^55^ In another approach, proteins might be first cut into peptides and the volume of each fragment is identified by the reduced nanopore current.^12,13,56−58^ When the peptidase is incorporated directly above the nanopore, proteins are identified at the single-molecule level.^14^ In a different approach, larger pores might be used to identify full-length, folded proteins as they enter the nanopore and are recognized by specific ionic current blockades.^17,31−33^ The latter may be extremely sensitive to small changes in the shape and charge of trapped proteins, allowing, for example, the detection of individual amino acid differences in a folded 65 kDa protein.^17^

In this work, we demonstrated folded protein analysis by biological YaxAB^34^ nanopores. The α-helical PFT has a 15 nm *cis*-entry and 3 nm *trans*-entry, describing the largest biological nanopore characterized for molecular analysis to date. Compared to other biological or solid-state nanopores, YaxAB showed unique properties. The conical shape of YaxAB allowed the entry and electrokinetic trapping of a large range of protein sizes. The lumen of the nanopore protrudes ∼11 nm above the lipid bilayer (Figure 1) and contains several fenestrations. Although such fenestrations allow the permeation of ions through the side of the nanopore, they contributed only a small part to the overall conductance of the nanopore. Moreover, theoretical models and MD simulations revealed that about 50% of the resistance is focused in the transmembrane region of the nanopore (*z* = 0–3.8 nm, Figure 1). These characteristics provide a unique protein recognition mechanism. Proteins showed an inverse correlation between the size of the protein and the residual current flowing through the nanopore. Most likely, this effect is due to deeper penetration of smaller proteins in the transmembrane region. Proteins smaller than the constriction region would translocate, and extended sampling of smaller proteins would require smaller YaxAB pores, as shown in recent work published during the peer review of this work.^59^

YaxAB might be particularly useful to characterize large proteins that typically cannot be addressed by other nanopores. We tested CRP (125 kDa), an important inflammatory biomarker whose concentration in blood is related to sepsis, which is one of the main causes of death in patients in intensive care units.^60,61^ Although the fragility of the lipid bilayer did not allow us to sample real biological samples, we showed here that CRP could be discriminated at clinically relevant concentrations (>2.5 mg/L), and that the presence of other human proteins in the sample seemed not to alter the CRP signature. Hence, if the stability of the lipid bilayer can be improved, for instance by using synthetic polymer membrane,^62,63^ the nanopore approach might be used in the clinic or at home for the real-time and continuous monitoring of protein biomarkers.

## Methods

### Materials

C-reactive protein (CRP, AG723), thrombin (CAS #9002-04-4), hemoglobin (CAS #54651-57-9), 6-cyclohexylhexyl β-d-maltoside (Cymal-6, CAS #228579-27-9), diphytanoyl-*sn*-glycero-3-phosphocholine (DPhPC, CAS #207131-40-6), and hexadecane 99% (CAS # 544-76-3) were obtained from Sigma/Merck. Phusion polymerase (M0530) was ordered from NEB. Streptavidin (21122), GeneJET Plasmid Miniprep Kit (K0503) GeneJET PCR Purification Kit (K0701), Phire Hot Start II DNA polymerase (F122S), T4 DNA ligase (EL0011), *Dpn*I (ER1701), *Nde*I (FD0583) and *Hind* III (ER0502) restriction enzymes, and High Select Top 14 Abundant Protein Depletion mini spin columns (A36369) were ordered from ThermoFisher Scientific. Other chemicals used were purchased from Carl-Roth. Sequencing was done by Macrogen and primers were acquired from Integrated DNA Technologies (IDT).

### Cloning of YaxA and YaxB Variants

The pRSET-A plasmids encoding YaxA and YaxB genes^34^ were kindly provided by Dr. Bastian Bräuning from Technische Universität München. The YaxA construct (PDB: 6EL1) contained additional mutations N17S, R150G, K250R, and S282G from the wild-type gene (YE1984^34^). The YaxB construct (PDB: 6EL1(34)) contained an additional mutation V284I from the wild-type gene (YE1985). The YaxA_Δ40_ variant was prepared by deleting the first 40 amino acids from the unstructured part of the WT sequence by ultramer PCR, using the primers in Table 2. The gene was amplified with Phire Hot Start II DNA polymerase (100 μL final volume, predenaturation at 98 °C for 30 s, 30 cycles of denaturation at 98 °C for 5 s, annealing at 54 °C for 15 s, and extension at 72 °C for 30 s) and purified using the GeneJET PCR Purification Kit. The YaxA_Δ40_ PCR product was digested with *Dpn*I (to remove circular template DNA of YaxA) for 2 h at 37 °C, and restricted-digested using the *Nde*I and *Hind* III restriction sites. The PCR purified product was ligated into *Nde*I and *Hind* III predigested pRSET-A vector with T4 ligase, and transformed into electrocompetent *E. cloni* cells (Lucigen). Plasmids were purified from acquired transformants with the GeneJET Plasmid Miniprep Kit and sent for sequencing (Macrogen). Successful clones were selected for further protein purification (Tables 3 and 4).

**Table 2:** Primers Used for Cloning YaxA_Δ40_

primer name	sequence 5′ > 3′
YaxA_Δ40_ forward	AGGAGGCCCATATGCATCACCATCACCATCACGAAAACTT- ATACTTCCAAAGTTCCGGCGGGATTTTTACTAAAGAGGAT
T7-term reverse	CTAGTTATTGCTCAGCGGTG

**Table 3:** DNA Sequences

His_6_-YaxA
ATGTCGTACTACCATCACCATCACCATCACGATTACGATATCCCAACGACCGAAAACCTGACGCGTTCGTCGGAGAACTTATACTTCCAAAGTGGTGGAACACAAACACAATTGGCTATTGATAATGTCTTGGCTAGTGCTGAAAGTACAATACAACTTAATGAGTTACCTAAAGTTGTTCTGGATTTTATCACCGGGGAGCAAACCAGTGTTGCGCGTTCCGGCGGGATTTTTACTAAAGAGGATTTAATTAACCTTAAACTTTATGTCAGAAAGGGGCTTTCATTACCAACCCGACAGGATGAAGTAGAGGCTTACCTTGGATATAAAAAAATAGATGTCGCTGGTCTTGAACCGAAAGATATTAAATTATTATTTGATGAAATTCATAATCATGCCTTAAATTGGAATGATGTGGAGCAGGCCGTATTACAACAAAGTTTGGATTTGGATATAGCAGCAAAAAACATCATCAGTACCGGCAATGAAATTATTAATCTGATAAATCAAATGCCAATAACCCTTCGTGTCAAAACCCTATTGGGTGACATTACAGATAAGCAGTTAGAGAATATCACTTATGAATCTGCTGATCATGAGGTAGCCTCAGCATTAAAAGATATTCTTGATGACATGAAAGGGGATATCAACAGGCATCAAACAACAACTGAGAATGTCAGGAAAAAAGTATCCGATTATAGAATCACCCTGACCGGCGGTGAGTTATCTTCGGGAGATAAAGTCAATGGGTTGGAACCACAGGTCAAAACAAAATACGACCTGATGGAAAAAAGCAATATGAGGAAATCAATAAAGGAATTAGACGAAAAAATAAAAGAGAAGAGACAGAGAATTGAGCAACTAAAGAAAGATTACGATAAGTTTGTCGGGTTGTCTTTTACTGGGGCCATAGGCGGTATAATAGCGATGGCTATTACTGGTGGGATCTTTGGTGCTAAAGCTGAAAACGCCAGAAAAGAAAAAAACGCATTAATTTCTGAAGTTGCTGAATTAGAAAGCAAAGTTAGCTCGCAAAGAGCATTACAAACTGCGTTAGAGGCACTTTCTCTGTCATTCAGTGATATTGGCATTCGAATGGTTGATGCCGAATCGGCGCTTAATCATTTGGATTTTATGTGGCTATCTGTTCTCAACCAAATTACTGAATCTCAGATACAGTTTGCGATGATTAACAATGCGTTGCGCCTGACTAGTTTTGTTAATAAATTCCAGCAGGTTATAACTCCATGGCAAAGTGTCGGAGATTCTGCCCGCCAGTTGGTGGATATATTTGATGAGGCAATAAAAGAATACAAAAAAGTGTATGGCTAA
His_6_-YaxA_Δ40_
ATGCATCACCATCACCATCACGAAAACTTATACTTCCAAAGTTCCGGCGGGATTTTTACTAAAGAGGATTTAATTAACCTTAAACTTTATGTCAGAAAGGGGCTTTCATTACCAACCCGACAGGATGAAGTAGAGGCTTACCTTGGATATAAAAAAATAGATGTCGCTGGTCTTGAACCGAAAGATATTAAATTATTATTTGATGAAATTCATAATCATGCCTTAAATTGGAATGATGTGGAGCAGGCCGTATTACAACAAAGTTTGGATTTGGATATAGCAGCAAAAAACATCATCAGTACCGGCAATGAAATTATTAATCTGATAAATCAAATGCCAATAACCCTTCGTGTCAAAACCCTATTGGGTGACATTACAGATAAGCAGTTAGAGAATATCACTTATGAATCTGCTGATCATGAGGTAGCCTCAGCATTAAAAGATATTCTTGATGACATGAAAGGGGATATCAACAGGCATCAAACAACAACTGAGAATGTCAGGAAAAAAGTATCCGATTATAGAATCACCCTGACCGGCGGTGAGTTATCTTCGGGAGATAAAGTCAATGGGTTGGAACCACAGGTCAAAACAAAATACGACCTGATGGAAAAAAGCAATATGAGGAAATCAATAAAGGAATTAGACGAAAAAATAAAAGAGAAGAGACAGAGAATTGAGCAACTAAAGAAAGATTACGATAAGTTTGTCGGGTTGTCTTTTACTGGGGCCATAGGCGGTATAATAGCGATGGCTATTACTGGTGGGATCTTTGGTGCTAAAGCTGAAAACGCCAGAAAAGAAAAAAACGCATTAATTTCTGAAGTTGCTGAATTAGAAAGCAAAGTTAGCTCGCAAAGAGCATTACAAACTGCGTTAGAGGCACTTTCTCTGTCATTCAGTGATATTGGCATTCGAATGGTTGATGCCGAATCGGCGCTTAATCATTTGGATTTTATGTGGCTATCTGTTCTCAACCAAATTACTGAATCTCAGATACAGTTTGCGATGATTAACAATGCGTTGCGCCTGACTAGTTTTGTTAATAAATTCCAGCAGGTTATAACTCCATGGCAAAGTGTCGGAGATTCTGCCCGCCAGTTGGTGGATATATTTGATGAGGCAATAAAAGAATACAAAAAAGTGTATGGCTAA
His_6_-YaxB
ATGTCGTACTACCATCACCATCACCATCACGATTACGATATCCCAACGACCGAAAACCTGTATTTTCAGGGAGCCGAAATAAGCACATTTCCACACAGTGGTCTGAGTTACCCAGACATTAATTTCAAAATTTTTAGCCAAGGCGTTAAAAATATATCTCATCTCGCCCAGTTTAAAACGACAGGCGTTGAAGTACTACAGGAAAAAGCACTACGAGTCAGTTTATATTCACAAAGGTTGGATGTTATTGTGCGAGAGTCATTGTCAAGTTTACAGGTTAAATTAGAAAATACTCTGGCTCTCACCTATTTTACCACTCTGGAAGAAATCGATGAGGCACTGATTAGTCAAGATATTGATGAAGAAAGTAAATCTGAAATGCGCAAAGAGCGCATTAATATTATTAAAAATCTATCTAATGACATTACTCAACTAAAGCAGTTGTTTATCGAAAAAACTGAGTTATTAGATAAGTCTTCCTCTGATCTGCATAACGTAGTGATTATTGAAGGAACCGACAAAGTCTTGCAAGCAGAACAATTACGTCAAAAACAACTGACAGAAGATATCGCTACCAAAGAACTGGAAAGAAAAGAGATTGAGAAAAAAAGAGATAAGATAATAGAAGCCTTGGATGTTATTCGCGAGCATAATCTGGTCGATGCATTCAAAGATCTTATCCCGACGGGCGAAAATTTAAGTGAATTAGATCTGGCTAAACCTGAAATAGAATTGCTTAAACAGTCATTAGAAATTACCAAGAAATTATTGGGGCAGTTTTCCGAGGGTTTAAAGTATATAGATTTAACTGACGCTCGGAAAAAGCTGGATAACCAAATAGATACCGCCTCCACCCGTTTAACCGAACTCAATCGCCAATTAGAGCAATCAGAGAAGTTAATTGCCGGTGTTAACGCGATTATTAAAATTGATCAGGAGAAAAGTGCTGTTGTTGTTGAGGCCGAAAAACTTAGCCGTGCATGGCATATTTTTATTCATGAGATAACAGCTCTACAAGGTACTTCACTGAATGAAGTTGAGCTATCGAAGCCACTAATAAAACAGCAGATCTATTTAGAGTCATTAATCAAACAGCTGATTTGA

**Table 4:** Protein Sequences

His_6_-YaxA, underlined = residues 1–40
MSYYHHHHHHDYDIPTTENLTRSSENLYFQSGGTQTQLAIDNVLASAESTIQLNELPKVVLDFITGEQTSVARSGGIFTKEDLINLKLYVRKGLSLPTRQDEVEAYLGYKKIDVAGLEPKDIKLLFDEIHNHALNWNDVEQAVLQQSLDLDIAAKNIISTGNEIINLINQMPITLRVKTLLGDITDKQLENITYESADHEVASALKDILDDMKGDINRHQTTTENVRKKVSDYRITLTGGELSSGDKVNGLEPQVKTKYDLMEKSNMRKSIKELDEKIKEKRQRIEQLKKDYDKFVGLSFTGAIGGIIAMAITGGIFGAKAENARKEKNALISEVAELESKVSSQRALQTALEALSLSFSDIGIRMVDAESALNHLDFMWLSVLNQITESQIQFAMINNALRLTSFVNKFQQVITPWQSVGDSARQLVDIFDEAIKEYKKVYG*
His_6_-YaxA_Δ40_
MHHHHHHENLYFQSSGGIFTKEDLINLKLYVRKGLSLPTRQDEVEAYLGYKKIDVAGLEPKDIKLLFDEIHNHALNWNDVEQAVLQQSLDLDIAAKNIISTGNEIINLINQMPITLRVKTLLGDITDKQLENITYESADHEVASALKDILDDMKGDINRHQTTTENVRKKVSDYRITLTGGELSSGDKVNGLEPQVKTKYDLMEKSNMRKSIKELDEKIKEKRQRIEQLKKDYDKFVGLSFTGAIGGIIAMAITGGIFGAKAENARKEKNALISEVAELESKVSSQRALQTALEALSLSFSDIGIRMVDAESALNHLDFMWLSVLNQITESQIQFAMINNALRLTSFVNKFQQVITPWQSVGDSARQLVDIFDEAIKEYKKVYG*
His_6_-YaxB
MSYYHHHHHHDYDIPTTENLYFQGAEISTFPHSGLSYPDINFKIFSQGVKNISHLAQFKTTGVEVLQEKALRVSLYSQRLDVIVRESLSSLQVKLENTLALTYFTTLEEIDEALISQDIDEESKSEMRKERINIIKNLSNDITQLKQLFIEKTELLDKSSSDLHNVVIIEGTDKVLQAEQLRQKQLTEDIATKELERKEIEKKRDKIIEALDVIREHNLVDAFKDLIPTGENLSELDLAKPEIELLKQSLEITKKLLGQFSEGLKYIDLTDARKKLDNQIDTASTRLTELNRQLEQSEKLIAGVNAIIKIDQEKSAVVVEAEKLSRAWHIFIHEITALQGTSLNEVELSKPLIKQQIYLESLIKQLI*

### Expression and Purification of YaxAB Monomers

The pRSET-A plasmids containing the YaxA or YaxB gene variants were transformed into *E. cloni* EXPRESS BL21(DE3) cells (Lucigen) by electroporation. The transformed cells were plated out on LB agar plates supplemented with 100 μg/mL ampicillin and grown overnight at 37 °C. The acquired colonies were inoculated into 2xYT medium supplemented with 100 μg/mL ampicillin. The expression culture was grown at 37 °C while being shaken at 200 rpm, until the optical density at 600 nm reached an OD_600_ of ∼0.8. To induce protein expression, 0.5 mM Isopropyl β-d-thiogalactopyranoside (IPTG) was added to the culture, which was then grown overnight at 25 °C while shaking at 200 rpm. The bacterial cells were harvested by centrifugation at 8000*g* for 15 min and stored at −80 °C.

The cell pellets were subjected to three freeze–thaw cycles to make the cells more susceptible to cell lysis. Each cell pellet, from 50 mL of cell culture, was resuspended in 20 mL of lysis buffer (50 mM Tris-HCl pH 8.0, 300 mM NaCl, 1 mM MgCl_2_, 10 μg/mL lysozyme, 0.2 U/mL DNase, with an additional 2 M urea for YaxA variants and one tablet of protease inhibitor EDTA-free per pellet of YaxB monomers) and incubated for 30 min at room temperature while shaking. The bacterial cells were disrupted by probe sonication (Brandson) at 30% output power for 3 × 60 s. Cell debris was removed by centrifugation at 4400*g* for 30 min at 4 °C. The supernatant was incubated for 15 min with 200 μL of Ni-NTA resin (Qiagen) at 4 °C while being rotated at 10 rpm. The incubated resin was loaded onto a gravity flow column (Bio-Rad) and washed with 10 mL of wash buffer (50 mM Tris-HCl pH 8.0, 300 mM NaCl, 10 mM imidazole). The protein was eluted from the Ni-NTA resin with 3x times 200 μL elution buffer (50 mM Tris-HCl pH 8.0, 300 mM NaCl, 100 mM Imidazole). The protein concentration was measured by Bradford assay and the monomers were stored at 4 °C until oligomerization.

### Oligomerization and Purification of YaxAB Oligomers

For oligomerization, the protein concentrations of both the YaxA and YaxB monomers were diluted to 1 mg/mL. Oligomerization is triggered by incubation of both YaxA and YaxB in a 1:1 ratio for 30 min at room temperature. It was shown that after oligomerization inactive oligodimers might be formed where the narrower *trans* sides of two pores stick to each other forming an hourglass-shaped oligomer.^34^ To separate such oligomers, 1.5% w/v 6-cyclohexylhexyl β-d-maltoside (Cymal-6) detergent was added to the solution for 30 min at 4 °C (longer incubation with Cymal-6 results in the dismantling of the pores back into monomers). Next, the incubation mixture was loaded on a size exclusion chromatography (SEC) column to lower the concentration of Cymal-6 and separate the unreacted monomers from single YaxAB pores. SEC was performed on an Akta pure chromatography system (Cytiva). YaxAB samples (500 μL) were loaded onto a Superose 6 10/300 GL SEC-column (Cytiva) pre-equilibrated with buffer A (25 mM HEPES pH 7.0, 150 mM NaCl, 0.05% w/v Cymal-6). Protein elution was monitored by measuring absorbance at a 280 nm wavelength. The first peak corresponded to the hourglass YaxAB oligodimers, the second peak corresponded to different oligomeric forms of YaxAB pores, and the third peak corresponded to the YaxA and YaxB monomers. Fractions of both sides of the main peak corresponding to YaxAB nanopores were collected separately, as they correspond to different oligomeric forms. The three fractions were sampled: one fraction at the center of elution peak (#18), one left (#17) and one right (#19) of this fraction. These fractions were aliquoted per 20 μL, flash frozen in liquid nitrogen, and stored at −80 °C until further use. Aliquots stored at −80 °C were stable for at least 2 years, with the exception of the triple mutants, which were stable for a couple of weeks. The center fraction (#18) contained predominantly YaxAB^80^ and was therefore used in electrophysiology experiments. The two other fractions contained larger (#17) or smaller (#19) pores (data not shown).

### Electrical Recordings in Planar Lipid Bilayers

The measuring setup consisted of a chamber containing two 500 μL compartments (*cis* and *trans)* separated by a 20 μm PTFE film with a central aperture of ∼100 μm diameter.^64^ A lipid bilayer was formed on the aperture by adding a drop of hexadecane [4% (v/v) in pentane] on to the *trans* side of the PTFE film directly above the aperture. Next, each compartment was filled with 400 μL of SDEX buffer (150 mM NaCl, 15 mM TrisHCl pH 7.5) and then two drops of 5 mg/mL DPhPC lipids. Ag/AgCl electrodes were inserted to each compartment: *trans* was the connecting electrode, *cis* was the ground electrode. By lowering and raising the buffer level in one compartment, a lipid bilayer was formed over the aperture. The bilayer was allowed to stabilize for 5 min. Prior to use, YaxAB aliquots were diluted ∼100× with buffer A, and diluted YaxAB was stable at 4 °C for weeks. A pipet tip (<0.1 μL) of diluted YaxAB was dipped into the buffer of the *cis* compartment, and generally a pore would insert within 10 min. The pore size was estimated by reading the current at a −35 mV potential. In SEC fraction #18 the current at −35 mV was usually between −60 and −100 pA, and predominantly −80 pA ± 10%. For protein capture experiments, the latter were used. All proteins were added to the *cis*, experiments were executed in triplicate. Measurements were conducted with a 50 kHz sampling frequency and a 10 kHz Bessel filter unless otherwise specified.

### Electrophysiological Data Recording and Analysis

All experimental nanopore data were recorded under a negative applied potential (−35 to −100 mV), using an Axopatch 200B patch clamp amplifier connected to a DigiData 1440 A/D converter (Axon Instruments), and using Clampex 10.7 software (Molecular Devices). *I*/*V* curves were taken from −100 to +100 mV with increment of 10 mV. Data recordings were made in gap-free setting or in a sweep protocol (−35 mV for 50 ms, +100 mV for 180 ms, and a measuring potential for 6 s). Recordings were analyzed with Clampfit 10.7 software (Molecular Devices). Only the experiments with depleted serum were additionally filtered with a Gaussian low-filter with 5 kHz cutoff prior to analysis. Open pore (*I*_O_) current was determined from Gaussian fits to all-point histograms with a bin width of 0.5 pA. Protein block currents (*I*_B_) were detected by the Single-Channel Search function in Event Detection. Residual current (*I*_RES_) was calculated as (*I*_B_/*I*_O_) × 100% for all events using an in-house Matlab script. Dwell time and standard deviation of *I*_B_ (σ_blockade_) of the blockades were also determined using the Single-Channel Search function. The σ_blockade_ was normalized by dividing it over the standard deviation of the open pore current (i.e., σ_blockade_/σ_open pore_). The *I*_O_ and σ_open pore_ were determined from a recording prior to the addition of analyte to *cis* (blank). At least 500 events at *I*_B_ were used for a cumulative histogram, to which a standard exponential could be fitted to determine the average dwell time (τ_off_). The release frequency (*f*_r_ = 1/τ_off_, in s^–1^) is equal to the off-rate (*k*_off_, s^–1^), i.e., *k*_off_ = 1/τ_off_. Similarly, at least 500 events at *I*_o_ were used for a cumulative histogram to determine the average interevent time (τ_on_). The capture frequency (*f*_c_ = 1/τ_on_, in s^–1^) was converted to the on-rate (*k*_on_, M^–1^ s^–1^) by *f*_c_ = *k*_on_ × [POI], where [POI] is the concentration of protein of interest as added to *cis*. The average on-rate per POI was calculated by least-squares regression. See also Figure S10.

### Ion Selectivity Determination by Reverse Potential Experiments

Reverse potentials were obtained using a similar electrophysiology setup as described above, with the exception of the following. Each compartment was filled with 400 μL of 300 mM NaCl and 15 mM TrisHCl, pH 7.5. Ag/AgCl electrodes were separated from the compartments by 1% agarose bridges containing a 3 M KCl solution. Upon pore insertion, the solution in *trans* was reduced to 78.6 mM NaCl in six steps. *I*/*V* curves from −100 to +100 mV were collected before and after buffer replacement. The resulting voltage at zero current is the reversal potential (*V*_r_). The ion selectivity (*P*_Na^+^_/*P*_Cl^–^_) was then calculated using the Goldman–Hodgkin–Katz eq 1, where [*a*_Na^+^/Cl^–^_ ]_*cis/trans*_ is the activity of the Na^+^ or Cl^–^ in the *cis* or *trans* compartment, *R* is the gas constant (8.3145 J mol^–1^ K^–1^), *T* is the temperature (298 K), and *F* is the Faraday constant (96 485 C mol^–1^).
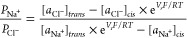
1The activity of ions was calculated by multiplying the molar concentration of the ion with the mean ion activity coefficients (0.7867 for 78.6 mM NaCl and 0.7224 for 300 mM NaCl, computed with linear regression from ref (65)).

### Calculating the Presence of Native CRP in Depleted Serum

Human serum (100 μL; Sigma-Aldrich, H4522) was depleted using High Select Top 14 Abundant Protein Depletion mini spin columns (ThermoFisher Scientific, A36369). The final volume was 300 μL; hence, the serum proteins were diluted 3x during depletion. YaxA_Δ40_B^80^ was used to detect serum proteins. Depleted human serum (2.5 μL) was added to *cis* (400 μL total volume), diluting the serum proteins 160x.

We measured YaxA_Δ40_B^80^ capturing depleted serum proteins for 627 s (>10 min). An event was annotated as “CRP” when its *I*_RES_, σ_blockade_, and dwell time values were within the mean ± 2σ as calculated for CRP in buffer [i.e., mean *I*_RES_ ± 2σ, mean σ_blockade_ ± 2σ, 10̂log(mean dwell time ±2σ), Table 1]. We found 46 events where the *I*_RES_ was similar to that of CRP in buffer. Among these, 21 events met the σ_blockade_-requirement, and 16 events met the dwell time-requirement (Figure 4a, Table S2). Six events (nos. 2, 9, 31, 32, 39, 46, Table S2) met *I*_RES_, dwell time, and σ_blockade_ requirements. This is in range with the expected number of CRP events present in serum, assuming the human serum was acquired from a person with a healthy CRP level (<3 mg/L).

Next, we measured YaxAB capturing depleted serum proteins for ∼2 min, titrated 20–80 nM CRP (final concentration) to the *cis* chamber, and again measured for ∼2 min. In total, we found 1040 events, of which the *I*_RES_ was similar to that of CRP in buffer (Table 1).

### General Molecular Dynamics Simulations Methods

All MD runs were carried out using NAMD^66^ software. The CHARMM36^67^ force field was employed to model lipid, protein, ions and TIP3P water molecules.^68^ Nonbonded fix corrections were applied for ions. The following standard parameters and methods are used: a time step of Δ*t* = 2.0 fs; periodic boundary conditions with hexagonal prism cell geometry; Particle Mesh Ewald-method^69^ with 1.0 Å spaced grid for long-range electrostatic interaction; a cutoff of 12 Å with switching distance of 14 Å was set for the short-range nonbonded interactions; Langevin thermostat (with damping 1/ps) and Nosé–Hoover Langevin piston pressure control (with period 100 fs and decay of 50 fs) was used for constant temperature and pressure simulations;^70^ all covalent bonds with hydrogen were kept rigid, using SETTLE^71^ for water molecules and SHAKE/RATTLE^72^ for the rest of the system.

### Pore Modeling and MD Setup

Both the YaxAB and YaxA_Δ40_B didecameric version of the pore were built starting from the YaxA and YaxB monomers X-ray crystal structures taken from the Protein Data Bank, PDB: 6EL1.^34^ The assembled pore structure is resolved by Cryo-EM and downloaded from the OPM database.^73^ The missing fragments for YaxA (Uniprot: A1JM51) and YaxB (Uniprot: A1JM52) monomers were rebuilt by using MODELLER^36^ software, and realigning each modeled subunits on each respective chain of the crystal structure 6EL1. Only the first best configuration for each chain is selected to rebuild the entire structure. In particular, an external loop in the head of YaxA subunit (residues D153-E167) and the N-terminus of YaxB were first modeled, for both the YaxAB and YaxA_Δ40_B systems identically. Then, only for the YaxA monomer version, the first 44 aa (amino acids) N-terminus were modeled using the entire YaxA sequence, obtaining a complete complex structure with the tails in random coil configuration, all oriented toward the pore axis and partially intertwined between them, see MD M0 model structure in Figure S2. Instead, the YaxA_Δ40_ subunits were modeled by removing the first 44 aa of the sequence at the N-terminus, i.e., by modeling only the I45-Y410 subsequence. The MD M0 configuration was also pre-equilibrated (see MD Equilibration section) to generate a second structure, MD M1, with the N-tails unfolded; see Figure S2. The determined YaxAB structures were embedded in a POPC membrane, solvated and neutralized with a 0.15 M NaCl water solution using VMD,^37^ using a protocol similar to the one reported in ref (74). The histidine residues are set as neutral in the HSE configuration. The resulting periodic box has a *x*–*y* hexagonal section apothem *a* = 135.5 Å and height *L*_*z*_ = 295 Å, for a total number of ∼1.8 million atoms.

### Pore Equilibration

After the modeling, we equilibrated one unique Δ40 configuration, while two different configurations (MD M0 and M1) were prepared for the YaxAB system. In the first one (MD M0) we directly equilibrated the complete simulation box built from the output of the protein modeling procedure, i.e., the one with the intertwined tails; see Figure S2. The second one (MD M1) is prepared by performing first a 500 ps MD run of the modeled structure in vacuum (without electrolyte) at high temperature (500 K), letting only the 40 aa N-tails free to move; the rest of the protein were fixed. The resulting “untwisted” structure is then embedded into a lipid membrane, solvated and neutralized as the other systems; see Figure S2. Then, the three systems (Δ40, M0, and M1) were equilibrated as in refs (44) and (74). In summary, the energy of the prepared systems were first minimized for 10 000 steps using the conjugate gradient method. Then a pre-equilibration of ∼1 ns is performed to let the lipid tails melt and the electrolyte relax: the temperature was increased from 0 to 300 K in 100 ps; external forces were applied to the water molecules to avoid their penetration into the membrane, while the backbone of the protein and the lipid heads were constrained to their initial positions by means of harmonic springs, with a spring constant *k*_b_ = 1 kcal/(mol Å^2^). After, another equilibration step with the protein released and without the harmonic constraints was performed for 20 ns to equilibrate the whole system.

### Ionic fluxes, Electroosmosis, and Total Electrical Force

The ionic fluxes and EOF were computed by nonequilibrium simulations, as previously done in other works.^44,74,75^ In brief, for each equilibrated system, we apply a uniform and constant electric field E = (0,0,E_*z*_) to the system perpendicular to the lipid bilayer, with *E*_*z*_ = Δ*V*/*L*_*z*_, mimicking an external voltage drop of ΔV across the membrane. Average currents and electroosmotic flow were calculated from the ions/water trajectories, after discarding a 10 ns transient (total length 130 ns). Errors were estimated using block average protocol, with block length 4 ns. The average total electrical force acting on the electrolyte, along the *z*-direction, represented in Figure S4, is calculated as *F*_el_ = *e*Σ_*i*_^*N*^*u*_i_/*μ*_*i*_, with *N* being the number of ions, *e* being the elementary charge, *u*_*i*_ being the average velocity (only component *z*) of the *i*th ion, and μ^+^ = 5.19 *m*^2^ (Vs)^−1^ and μ^–^ = 7.91 *m*^2^ (Vs)^−1^ being the ionic mobility of sodium and chloride ions, respectively.^40^ The expression for *F*_el_ can be computed by considering a charged particle under an electric field in steady-state motion where *F*_el_ = −*F*_drag_. For a spherical particle, *F*_drag_ = −*u*_*i*_/*μ*_*i*_.

### Pore Resistance and Hindrance Estimation

The pore resistance as a function of the electrolyte accessible area profile along the pore axis z is computed as previously described,^38^ from equilibrium MD simulations (without any applied external electric field E). In brief, the total resistance of the nanopore can be written as Ω = *Ω*_acc_ + ∫ ω(*z*) d*z*, with Ω_acc_ being access resistance and ω(*z*) = (σ*A*(*z*))^−1^ being resistance per unit length (Figure S4), where σ is the conductivity of the electrolyte and *A*(*z*) the available conducting section. To estimate *A*(*z*), we computed a discretized 3D map *M*(*x*,*y*,*z*) of the average occupancy of the electrolyte inside the pore. From each plane of *M*, we obtain an available conducting section *A*(*z*). To compute *M*(*x*,*y*,*z*), an additional filter is applied to the algorithm presented in ref (38), since the YaxAB pore does not have a completely closed geometry. Indeed, lateral channels are present connecting the interior and the exterior of the nanopore; see Figure S4. However, the conductivity of such lateral openings can, in first approximation, be neglected. Indeed, despite the total available area through the lateral channels (and length) would allow for about 1 nS of total conductivity, for all the presented cases there cannot be any significant voltage drop across these lateral channels, since all the voltage drop (induced by an external electric field) will be focused into the transmembrane part of the pore. To have a significant current flowing through the lateral fenestration, it would be required that the largest, outermost part of the pore is highly hindered (>80% or the available area), so that the voltage drop across the lateral channels increases. We estimated that just in the CRP case it is hypothetically possible that about 10% of the total current would flow through the lateral fenestrations. In the other cases the lateral current will be about 5%. So, for each plane *z*_*i*_, we filtered out all the cells exceeding a certain radius r_i_, setting the corresponding *M*(*x*,*y*,*z*) to zero: 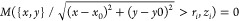
The radius *r*_*i*_ corresponds to the outermost radius (centered on the pore axis) having an average occupancy *M̲*(*r*_*i*_,*z*_*i*_) < 0.5 *m*_bulk_, with *m*_bulk_ being the average occupancy of the 0.15 M NaCl water solution in a 1 × 1 × 1 Å^3^ cell. The average occupancy *M̲*(*r*_*i*_,*z*_*i*_) is computed by averaging over all of the cells of *M*(*x*,*y*,*z*) corresponding to the radius *r*_*i*_. The effective radius in Figure 2 is computed from available area *A*(*z*) = π*r*(*z*)^2^.

The same protocol is used for computing the resistance in the presence of the different proteins inside the nanopore; the relative resistance profile plots are reported in Figure S22. From Ohm’s law Δ*V* = *IR*, we defined the quantity *I*_res_*MD*[%] = 100 × Ω_Δ40_/Ω_*b*_ in analogy to the experimental residual current.

### Inserted Protein Modeling and Steered Molecular Dynamics

Here, the word “protein” refers to the four CRP, HG, SA, and BT molecules, while “nanopore” is used to indicate the YaxA_Δ40_B pore.

Proteins are modeled starting from the PDBs, CRP 1GNH, SA 6J6J, HG 1B86, BT 1MKW, completing the missing fragments, where needed, using the MODELLER^36^ software, as done for the nanopore. Each modeled structure was first minimized in vacuum with 5000 steepest descent steps followed by 5000 MD steps (time step 0.5 ps). For each of the four modeled structures, three MD systems were prepared: Ox, Oy, and Oz, obtained by merging the protein in different orientations with the previously modeled nanopore. For each system, the center of mass of the protein was positioned at about 1 nm outside the center of the larger pore opening, in position (0, 0, 160), see the reference system of Figures 3a and S22. Instead, the principal inertial axis of the protein was aligned to the x, y, or *z* axis for the three different Ox, Oy, Oz systems, respectively. A slab of water having half of the height of the protein was also added to the system. The resulting MD box was finally ionized at 0.15 M. Systems prepared using VMD^37^ and its plugins.

For each prepared system, a 2 ns NPT simulation (*P* = 1 atm, *T* = 310 K, variable time step), keeping the center of mass and orientation of the protein constrained, was performed to equilibrate the system. Then, the protein was pushed inside the nanopore lumen by applying a constant and homogeneous force *F*_*i*_ = (0,0,*f*_*z,i*_) on each heavy protein atom (not hydrogens) with a total applied force *F*_*z*_ = Σ_*i*_*f*_*z,i*_ = −690 pN (Steered Molecular Dynamics, SMD). The simulations lasted for 35 ns. The average steady state positions of the center of mass *z̅*_COM_ and relative pore hindrance (see previous section Resistance and Pore Hindrance Estimation) of the inserted proteins was then computed over the last 12 ns of simulations.

## Data Availability

All data and corresponding analysis generated in this study have been deposited in the Zenodo database under DOI: 10.5281/zenodo.8039199.

## Supporting Information Available

Tables containing the biophysical property values of the proteins analyzed and a list of the CRP event detected in serum; figures showing additional information about the structure of YaxAB, SDS of the protein tested, details about MD simulations, electrical properties of the nanopore, and detection of proteins with nanopores of different size and composition (PDF)
