# Supplementary material for: Protein Sizing with 15 nm Conical Biological Nanopore YaxAB
Source: ACS Nano. 2023 Jul 17;17(14):13685–99. doi: 10.1021/acsnano.3c02847 (PMC10373527; doi:10.1021/acsnano.3c02847)
Supplement: Supplementary file 1 — nn3c02847_si_001.pdf [file nn3c02847_si_001.pdf]

Supporting Information

Protein sizing with 15-nm conical biological nanopore YaxAB

*Sabine Straathof[1#], Giovanni Di Muccio[2+#], Maaruthy Yelleswarapu[1#], Melissa Alzate Banguero[2++], Carsten Wloka[1], Nieck Jordy van der Heide[1], Mauro Chinappi[2] and Giovanni Maglia[1]**

[1]Groningen Biomolecular Sciences & Biotechnology Institute, University of Groningen, 9747 AG Groningen, The Netherlands
[2]Department of Industrial Engineering, University of Rome Tor Vergata, Via del Politecnico 1, 00133 Rome, Italy
[+]Present, Department of Mechanical and Aerospace Engineering, Sapienza Università di Roma, 00184 Rome, Italy
[++]Present, Laboratoire de Physique et d'Étude des Matériaux, ESPCI Paris, PSL Université, 75005, Paris, France

[#] *S.S., G.D.M. and M.Y. contributed equally to this paper*

*Email: Giovanni.maglia@rug.nl

*Email: Mauro.chinappi@uniroma2.it

Keywords

nanopores, electrophysiology, folded protein analysis, single-molecule, electroosmosis

Table of content

Figure S1. Comparing dimensions of large biological nanopores.
Figure S2. Di-decameric YaxAB molecular modeling.
Figure S3. Experimental I-V curves of frequently observed $YaxA_{\Delta40}B$ assemblies.
Figure S4. Conductance estimation for the di-decameric YaxAB and $YaxA_{\Delta40}B$ nanopore.
Figure S5. Isolated charged residues in $YaxA_{\Delta40}B$.
Figure S6. MD simulated EOF and ion selectivity for mutated $YaxA_{\Delta40}B$.
Figure S7. The reverse potential (V_r) of YaxAB pores.
Figure S8. Current distributions for the $YaxA_{\Delta40}B_{RRR}$ and $YaxA_{\Delta40}B_{NNN}$.
Figure S9. SDS-PAGE of CRP, SA, HG, BT.
Figure S10. Definition of nanopore event parameters.
Figure S11. CRP trapping in $YaxA_{\Delta40}B$ with different conductances.
Figure S12. Unmodified $YaxAB^{80}$ capturing proteins.
Figure S13: Representative traces of protein capture by $YaxA_{\Delta40}B^{80}$ in triplicate
Figure S14: CRP capture in $YaxA_{\Delta40}B^{80}$ at increasing voltage.
Figure S15: HG capture in $YaxA_{\Delta40}B^{80}$ at increasing voltage.
Figure S16: SA capture in $YaxA_{\Delta40}B^{80}$ at increasing voltage.
Figure S17: BT capture in $YaxA_{\Delta40}B^{80}$ at increasing voltage.
Figure S18: BT trapping in $YaxA_{\Delta40}B$ with different conductances.
Figure S19. Voltage-dependent capture of folded proteins in $YaxA_{\Delta40}B^{80}$.
Figure S20. Hemoglobin capture at different voltages.
Figure S21: Hemoglobin sublevel analysis as detected by $YaxA_{\Delta40}B^{80}$.
Figure S22. Steered MD simulations and hindrance estimation of the CRP, SA, HG and BT proteins inside the di-decameric $YaxA_{\Delta40}B$.
Figure S23. Concentration-dependent capture of folded proteins in $YaxA_{\Delta40}B^{80}$.
Figure S24: Improving resolution of mixed sample with $YaxA_{\Delta40}B$ pores with different conductance
Figure S25. Full $\sigma_{blockade\ vs\ IRES}$ scatterplot of CRP events in presence depleted human serum.
Figure S26. Interpreting potential native CRP events in depleted serum.
Figure S27. Comparing CRP in buffer and CRP titrated on top of depleted serum

Table S1. Biophysical values of the proteins: experimentally fitted vs. theoretical.
Table S2. Potential native CRP events in depleted serum.

Supplementary Note. Note on the fit of the capture frequency of **Figure S23**.

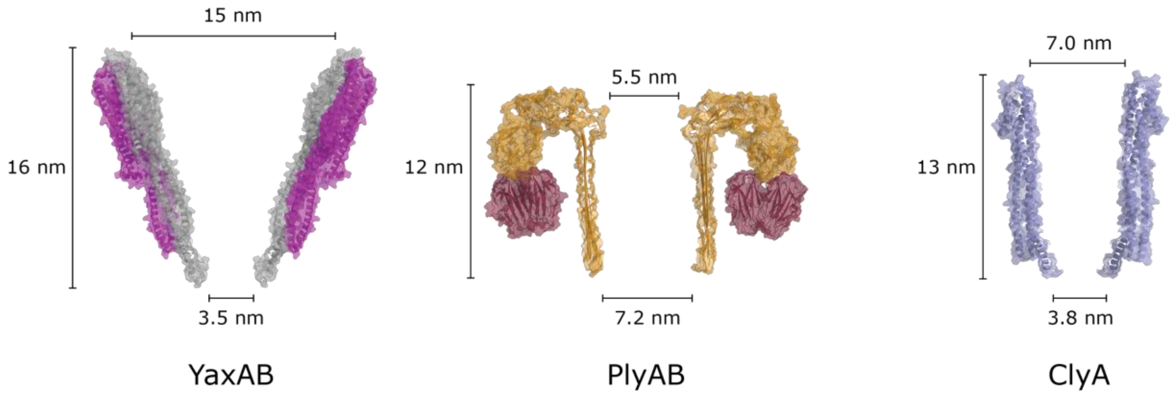

Figure S1. **Large biological nanopores for protein characterisation.** Left, YaxAB (PDB: 6EL1), with YaxA in purple and YaxB in grey. Middle, PlyAB[1], with PlyA in red and PlyB in orange. Right, ClyA (PDB: 2WCD) in pale blue. The figure was generated in Pymol and the structures are to scale.

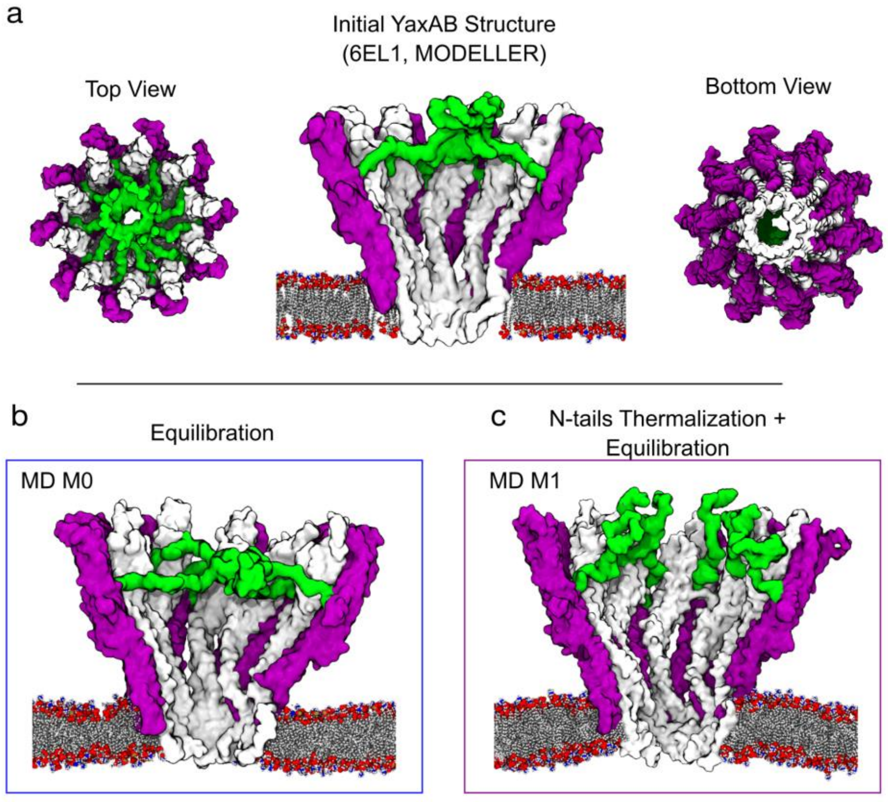

d

```
>6EL1_1 | Chains A,B,C,D,E,F,G,H,I,J | YaxA | Yersinia enterocolitica (630)
         10|       20|       30|       40|       50|       60|       70|       80|       90|      100|
TQTQLAIDNVLASAESTIQLNELPKVVLDFITGEQTSVARSGGIFTKEDLINLKLYVRKGLSLPTRQDEVEAYLGYKKIDVAGLEPKDIKLLFDEIHNHA
LNWNDVEQAVLQQSLDLDIAAKNIISTGNEIINLINQMPITLRVKTLLGDITDKQLENITYESADHEVASALKDILDDMKGDINRHQTTTENVRKKVSDY
RITLTGGELSSGDKVNGLEPQVKTKYDLMEKSNMRKSIKELDEKIKEKRQRIEQLKKDYDKFVGLSFTGAIGGIIAMAITGGIFGAKAENARKEKNALIS
EVAELESKVSSQRALQTALEALSLSFSDIGIRMVDAESALNHLDFMWLSVLNQITESQIQFAMINNALRLTSFVNKFQQVITPWQSVGDSARQLVDIFDE
AIKEYKKVYG

>6EL1_2 | Chains K,L,M,N,O,P,Q,R,S,T | YaxB | Yersinia enterocolitica (630)
GAEISTFPHSGLSYPDINFKIFSQGVKNISHLAQFKTTGVEVLQEKALRVSLYSQRLDVIVRESLSSLQVKLENTLALTYFTTLEEIDEALISQDIDEES
KSEMRKERINIIKNLSNDITQLKQLFIEKTELLDKSSSDLHNVVIIEGTDKVLQAEQLRQKQLTEDIATKELERKEIEKKRDKIIEALDVIREHNLVDAF
KDLIPTGENLSELDLAKPEIELLKQSLEITKKLLGQFSEGLKYIDLTDARKKLDNQIDTASTRLTELNRQLEQSEKLIAGVNAVIKIDQEKSAVVVEAEK
LSRAWHIFIHEITALQGTSLNEVELSKPLIKQQIYLESLIKQLI
```

Figure S2. Di-decameric YaxAB molecular modeling. a) Initial structure of the modeled YaxAB, obtained by merging together the structures of all the modeled chains of the PDB structure 6EL1 (MODELLER[2] software, see Methods), embedded into a POPC lipid bilayer. The YaxA monomers are represented in purple and the N-terminal tails of YaxA are in green, while the YaxB monomers are represented in white. **b)** Equilibrated structure without the N-tails pre-thermalization (see Methods) and **c)** after the "N-tail thermalization". The latter (MD M1, purple rectangle) system is the same reported in Fig. 1 of the main manuscript. Pictures are realized with VMD[3] software. **d)** Amino acid sequence (PDB: 6EL1 FASTA) of the YaxAB complex. In red is highlighted the part of the sequence that was deleted in the YaxA$_{\Delta 40}$ mutant. This region has a net charge of -3 (#[R or K] - #[D or E]) at pH 7.5.

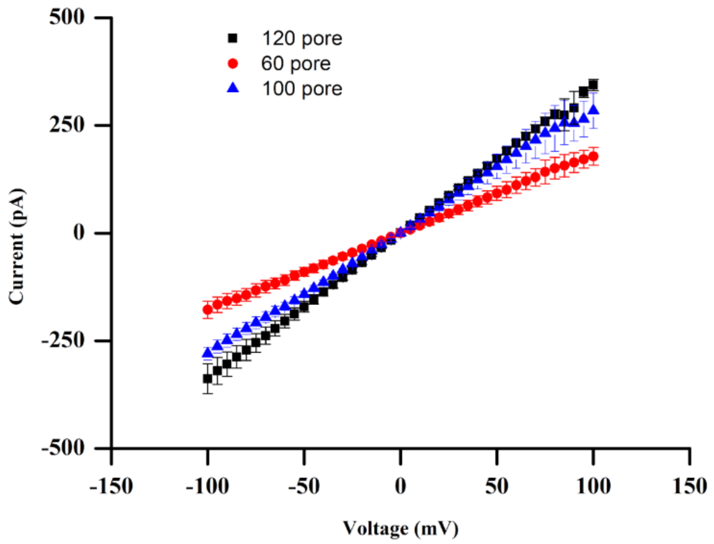

Figure S3. Experimental I-V curves of frequently observed YaxA$_{\Delta 40}$B assemblies. Pores used with an open pore current at -35 mV of 60, 100 and 120 pA (-35 mV), represented in red, blue and black, respectively. The I-V curve for the YaxA$_{\Delta 40}$B^{80} is reported in **Fig. 2a** of the main text. Measurements were conducted with a 10 kHz sampling rate and a 2 kHz Bessel filter in 150 mM NaCl, 15 mM Tris-HCl pH 7.5.

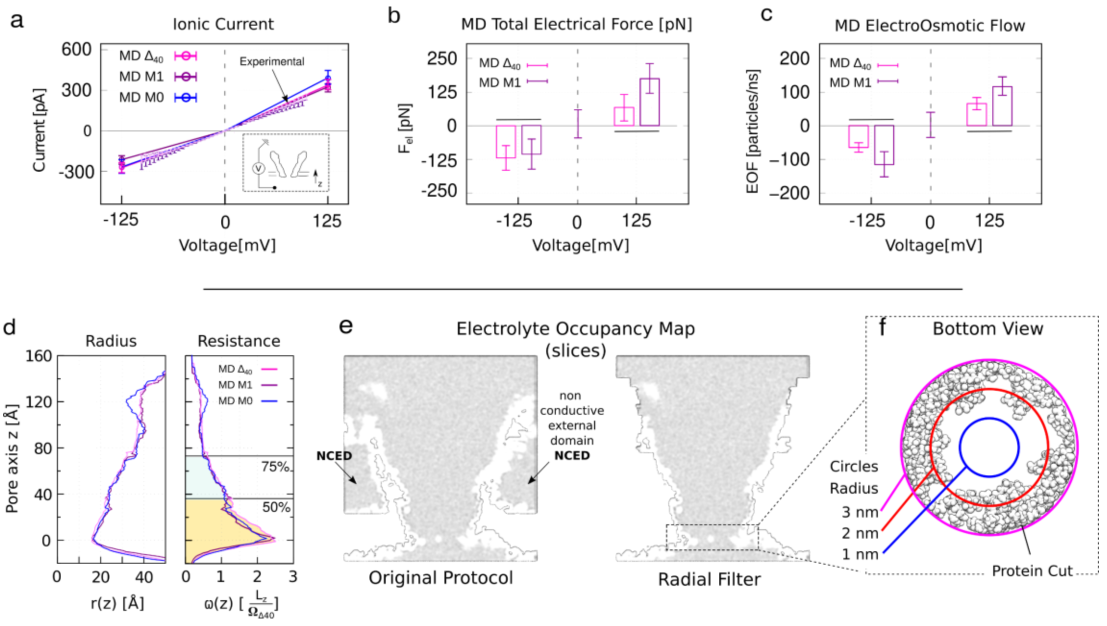

Figure S4. Conductance estimation for the di-decameric YaxAB and YaxA$_{\Delta40}$B nanopore. a) Ionic currents estimated by MD simulations at ±125 mV applied voltage. The MD simulation showed that the di-decameric YaxAB (MD M0 and M1 - M0 and M1 refer to the systems shown in **Figure S2**) and YaxA$_{\Delta40}$B (MD Δ40) systems exhibit indistinguishable currents. The ionic currents computed by MD are divided by a factor ν=1.5 since the 1M KCl electrical mobility, in TIP3P water (CHARMM force field), is ν times larger than the experimental one.[4] **b**) Total electrical force, expressed as the force exerted on the dissolved ions by the applied electric field, and **c**) EOF, computed by non-equilibrium MD simulations at positive and negative applied bias, along the z direction. **d)** Effective radius of the inner electrolyte volume and related resistance-per-unit-length ω(z), along the pore axis z. ω(z) is normalized by the average resistance-per-unit-length of the YaxA$_{\Delta40}$B system, $\omega_{\Delta40} = \frac{\Omega_{\Delta40}}{L_z} = 26 \times 10^{12}\ \Omega/nm$, with $\Omega_{\Delta40}$ total resistance and $L_z = 15\ nm$ length of the pore. The area indicated in yellow and light green represents 50% and 75% of the cumulative resistance, respectively, for both the decameric YaxAB and YaxA$_{\Delta40}$B systems. **e)** Slices of the 3D occupancy map computed by using the original protocol described in ref.[5] and the modified one with the “radial filter”, as described in Methods section Resistance and Pore Hindrance Estimation. Gray areas correspond to the areas more occupied by the electrolyte (electrolyte bulk fractional occupancy ψ = 0.42 Å^{-3}), while the white areas are less occupied. In the left panel it can be noted that the YaxAB (being composed of separated alpha-helices) does not present a strictly closed internal domain. We applied a radial filter as described in Methods, to define an internal reasonable conductive volume (the one represented in the right panel). **f)** Bottom view of the molecular system, zoom on the constriction region. The white (VDW balls) structure represents the atoms of the protein whose distance < 3 nm from the center. The colored circles display three representative radii of 1, 2 and 3 nm in blue, red and pink, respectively.

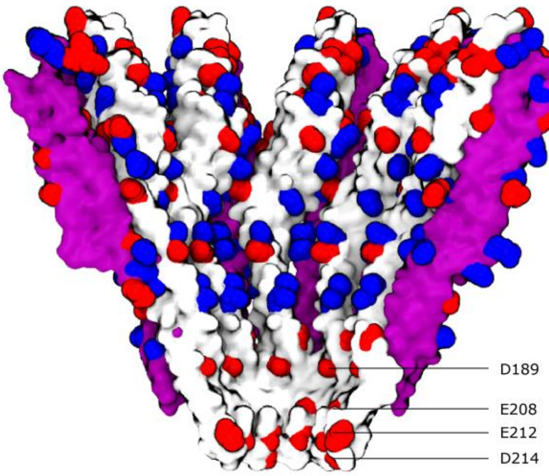

Figure S5. Isolated charged residues in YaxA$_{\Delta40}$B. Section of PDB structure 6EL1, showing the surface representation of YaxA (purple) and YaxB (white) protonomers; red and blue spheres represent the sidechain of the "isolated" charges residues (Arg, Lys, Asp, Glu) that don't lie within a Debye length (8 Å at 0.15M) of any opposite charged residues; in red are the acidic and in blue the basic isolated residues.

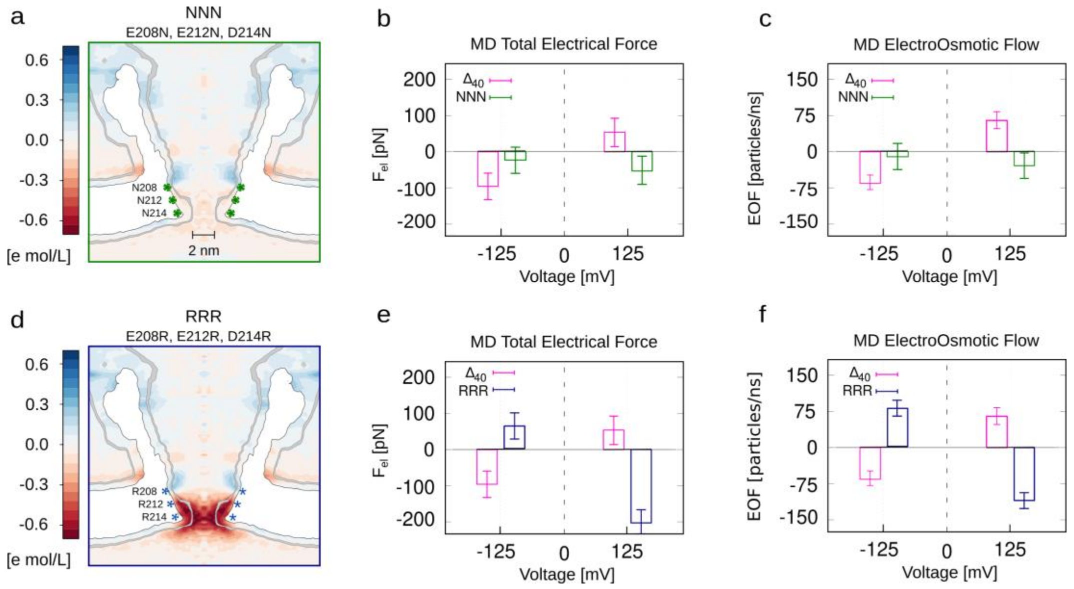

Figure S6. MD simulated EOF and ion selectivity for mutated YaxA$_{\Delta 40}$B. The Δ40 label in the different panels refers to the di-decameric YaxA$_{\Delta 40}$B data, shown in **Fig. 2** of the main text. For the NNN system (**panels a-c**), the charge of the constriction region was neutralized by mutating the three rings of acidic (negatively charged) residues in YaxB, indicated by the asterisks into three neutral rings of Asparagine (N). For the RRR system (**panels d-f**) the charge of the constriction region in YaxB was inverted by mutating the three rings of acidic (negatively charged at pH 7) residues indicated by the asterisks into three basic (positively charged at pH 7) rings of Arginine (R). The net charge density maps display the radial average of the charge density computed over the dissolved ions in the electrolyte at equilibrium (ΔV=0). The total electrical force is the force exerted by the external electric field on the dissolved ions, see MD Methods. The EOF is the average flow of water molecules, computed from the average velocities of the water oxygen atoms.

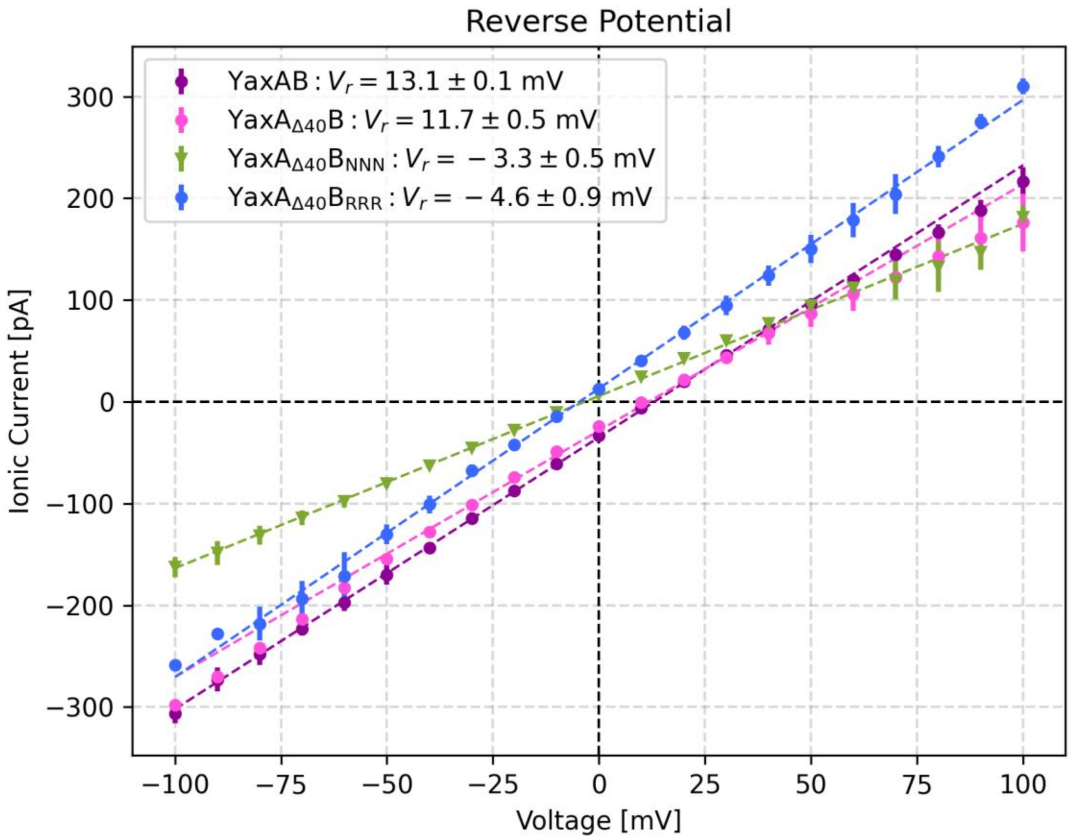

Figure S7: The reverse potential (V_r) of YaxAB pores. After pore insertion in symmetrical salt conditions (300 mM NaCl, 15 mM Tris, pH 7.5 in *cis* and *trans*), the *trans* compartment was perfused six times to 78.6 mM NaCl, 15 mM Tris pH 7.5 and an I/V-curve was recorded. The V_r was determined to be -3.3 ± 0.5 mV for YaxA$_{\Delta 40}$B$_{NNN}$^{40} and -4.6 ± 0.9 mV YaxA$_{\Delta 40}$B$_{RRR}$^{80} , respectively (see **Figure S8** for the definitions). We also include the YaxA$_{\Delta 40}$B^{80} and YaxAB^{80} experiments for comparison. Using the Goldman–Hodgkin–Katz equation (see Methods), the resulting selectivity P_{Na+}/P_{Cl-} are 0.80 ± 0.04, 0.72 ± 0.01, 2.46 ± 0.22 and 2.60 ± 0.09 for YaxA$_{\Delta 40}$B$_{NNN}$^{*40}, YaxA$_{\Delta 40}$B$_{RRR}$^{80}, YaxA$_{\Delta 40}$B^{*80} and YaxAB^{80}, respectively. The activity coefficients used for are 0.7867 for 78.6 mM NaCl and 0.7223 for 300 mM NaCl. Data was recorded at 50 kHz sampling rate and 10 kHz Bessel filter.

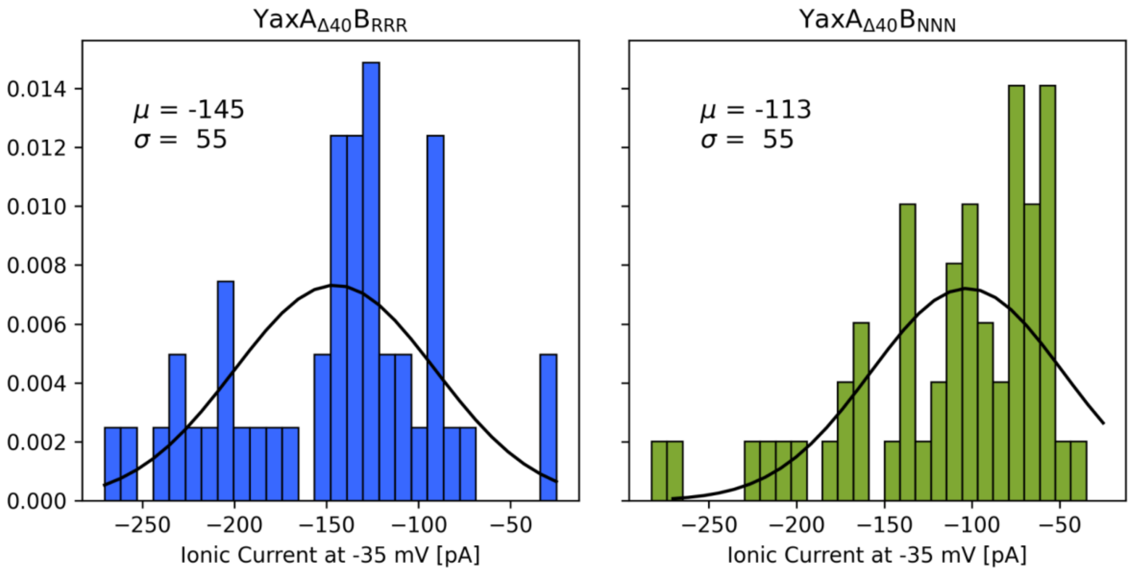

Figure S8: Current distributions for the $YaxA_{\Delta40}B_{RRR}$ and $YaxA_{\Delta40}B_{NNN}$. For the RRR system (left), N=46 pores from 8 independent recordings were included. For the NNN system (right), 56 pores from 3 independent recordings were included. Gaussian fits (mean µ and standard deviation σ) of the distributions are reported to guide the eye. Compared to the $YaxA_{\Delta40}B^{80}$ pore, [I_O = -145.2 ± 6.1 pA (N=3) in 0.3 M NaCl] we can assume that the RRR mutant main assembly is similar to the main assembly of $YaxA_{\Delta40}B^{80}$. Instead, the NNN pore presents a main peak around -80 pA at 0.3 M NaCl, a value that is about half the one measured for $YaxA_{\Delta40}B^{80}$ pore at the same salt concentration. The lower current value of the NNN mutant possibly corresponds to a smaller oligomeric assembly. Data was collected at 50 kHz sampling rate and 10 kHz Bessel filter in 300 mM NaCl, 15mM Tris-HCl, pH 7.5. Notice that the ionic strength here is double the amount of salt (150 mM NaCl) compared to **Fig. 1** of main text.

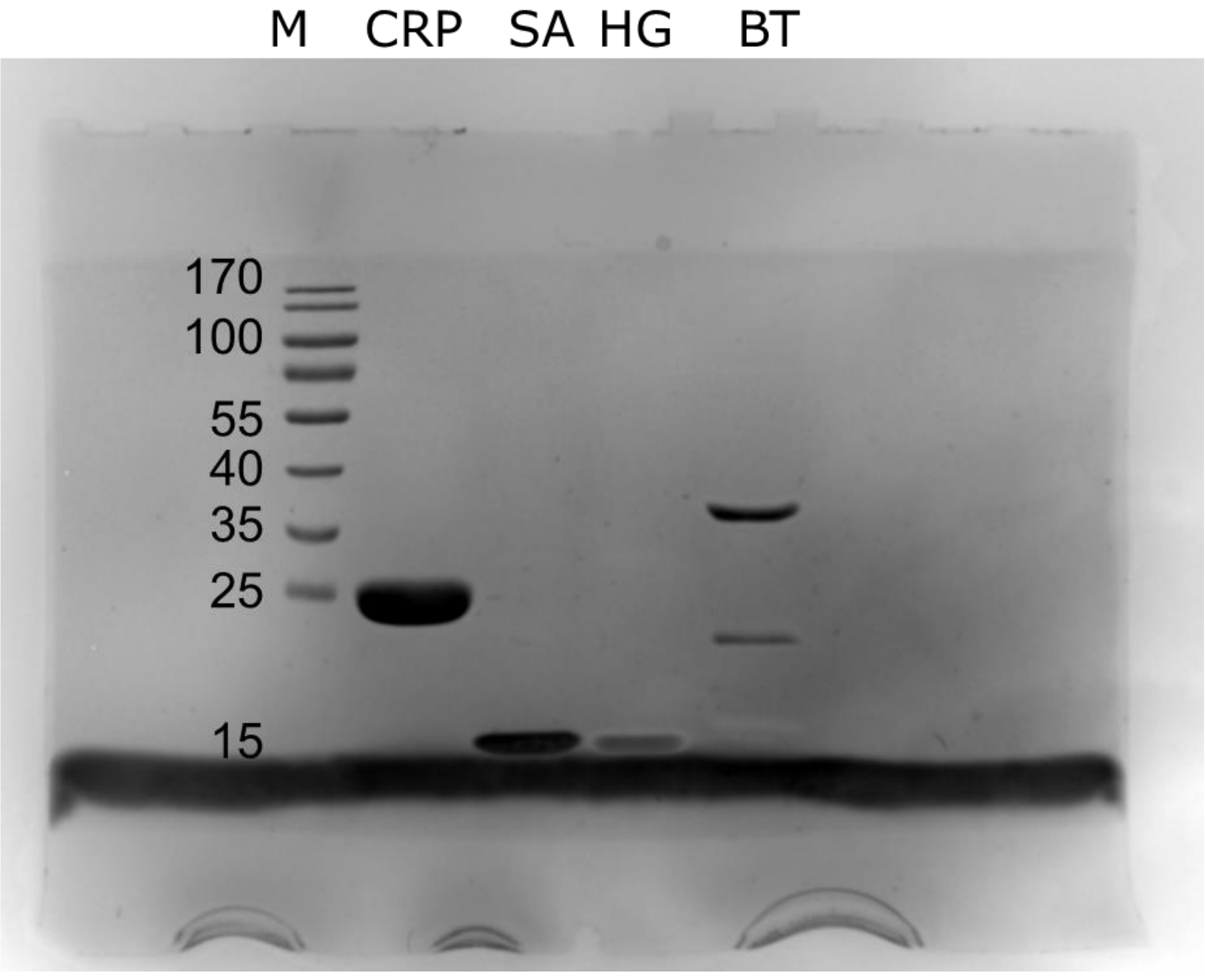

Figure S9: 12% SDS-PAGE of CRP, SA, HG, BT. CRP (125 kDa) is pentameric; SA (53 kDa) is tetrameric; HG (64 kDa) is heterotetrameric ($\alpha_2\beta_2$); BT (35 kDa) is heterodimeric (αβ). Marker (M, in kDa) is PageRuler Prestained Protein Ladder (Thermofisher). The small contamination of BT at ~20 kDa is not observed in electrophysiology traces, presumably because it is not captured or translocates faster than the sampling frequency and is therefore insignificant.

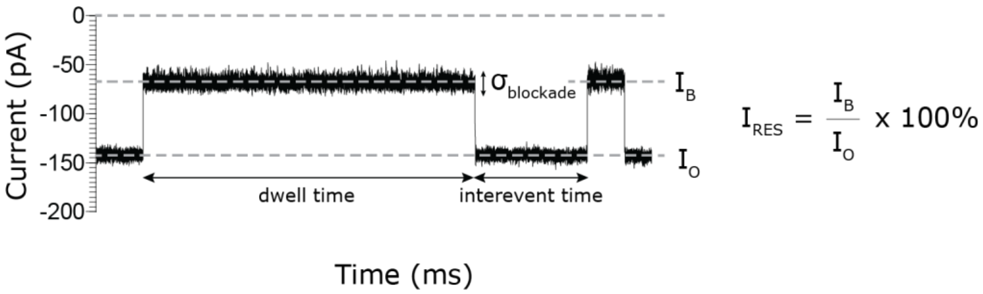

Figure S10: Nanopore event parameters. The open pore current (I_O, pA) is the average ion flux through a given single nanopore under a given potential. The blockade current (I_B, pA) is the average (reduced) ion flux when an analyte enters the pore. The I_B is a temporary interruption of the I_O. The ratio between the I_B and I_O (residual current, I_{RES}= (I_B/I_O) × 100%) reflects the remaining ion flux during the event. The $\sigma_{blockade}$ (pA) is the standard deviation in the signal of I_B. The $\sigma_{blockade}$ is normalized by the $\sigma_{blockade}/\sigma_{open\ pore}$. The dwell time (ms) is the duration of an event. The interevent time (ms) is the duration in between events. The parameters were calculated by Clampfit Software version 10.7 (Molecular Devices) with the Single-Channel Search function (see Methods). The average dwell time (τ_{off}) can be described as the release frequency ($f_r = k_{off} = 1/\tau_{off}$, s^{-1}) and is characteristic to the analyte and voltage. The average time in between events (τ_{on}) can be described as the capture frequency ($f_c = 1/\tau_{on}$, s^{-1}) and is dependent on concentration ($f_c = k_{on}$×[POI], s^{-1}) and voltage.

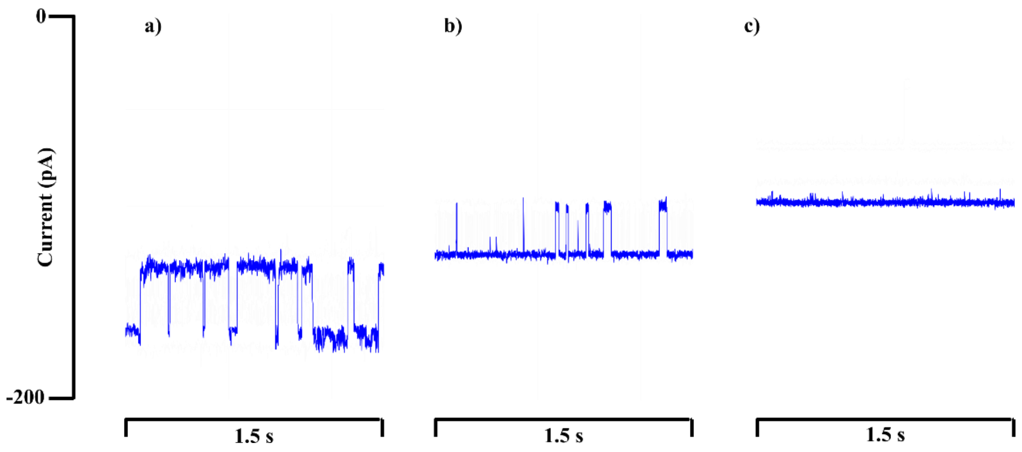

Figure S11. CRP trapping in YaxA$_{\Delta40}$B with different conductance. a) YaxA$_{\Delta40}$B^{120}; **b)** YaxA$_{\Delta40}$B^{80}; and **c)** YaxA$_{\Delta40}$B^{60}. The superscripts indicate the approximate conductance of the nanopore at -35 mV. Measurements were performed in 150 mM NaCl, 15 mM Tris-HCl pH 7.5, at -60mV applied potential. Data was collected at 10 kHz sampling rate and 2 kHz Bessel filter.

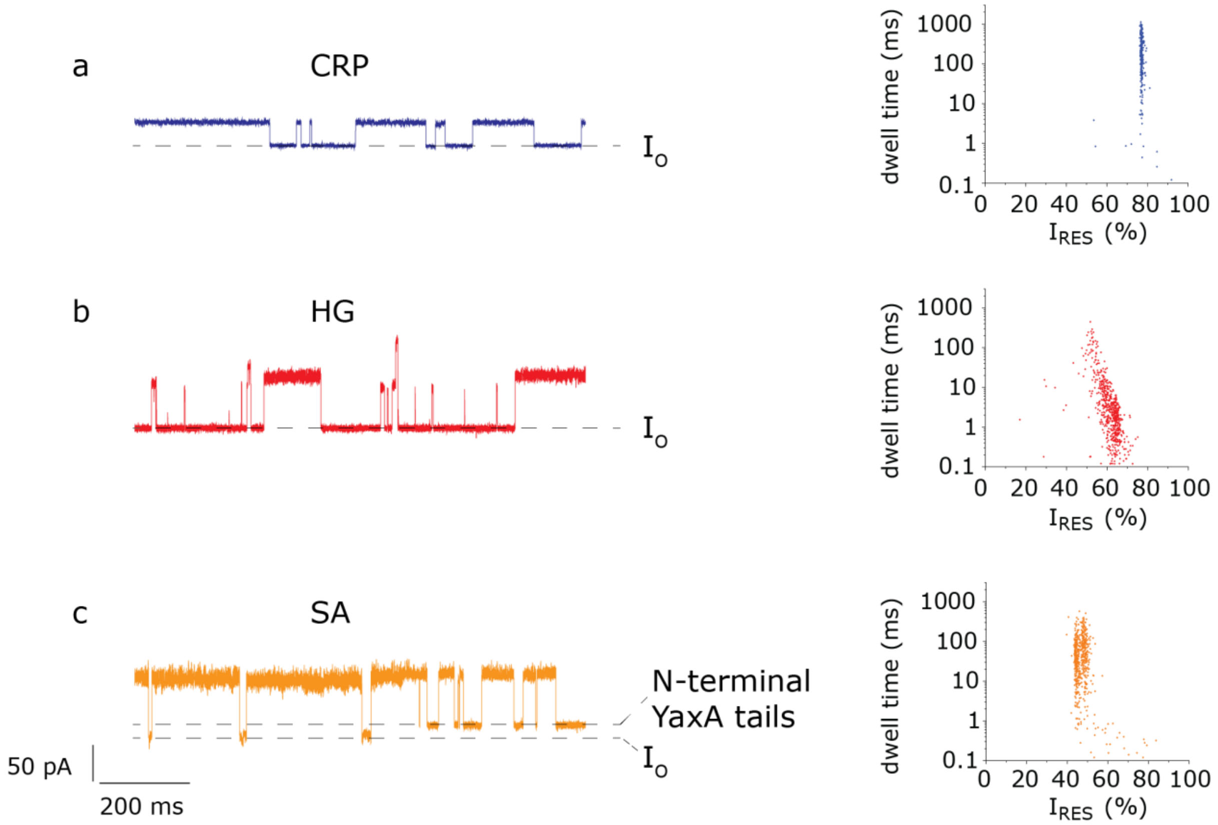

Figure S12. Protein capture by WT-YaxAB[80]. Representative traces and accompanying dwell time vs I_{RES} scatterplots of **a)** CRP; **b)** HG; **c)** SA. N-terminal tails (residues 1-40) of YaxA do not interfere with protein capture. Occasionally, we observed that the open pore current (I_O) changed during the experiment (c), possibly an effect mediated by the N-terminal tail. We therefore continued with a truncated YaxA (YaxA$_{\Delta 40}$B) to ensure stable open pore current. Experiments were conducted at 150 mM NaCl, 15 mM Tris-HCl, pH 7.5, with 200 nM protein in *cis*, and -75 mV applied potential. Sampling frequency was set at 50 kHz for all experiments, with Bessel filter at 2 kHz (a), or at 10 kHz (b and c). Traces in b and c were additionally filtered with 5 kHz low-pass Gaussian filter for visualization. Scatterplots contain n = 281 (a) or n = 600 datapoints (b and c) from N=1 pore.

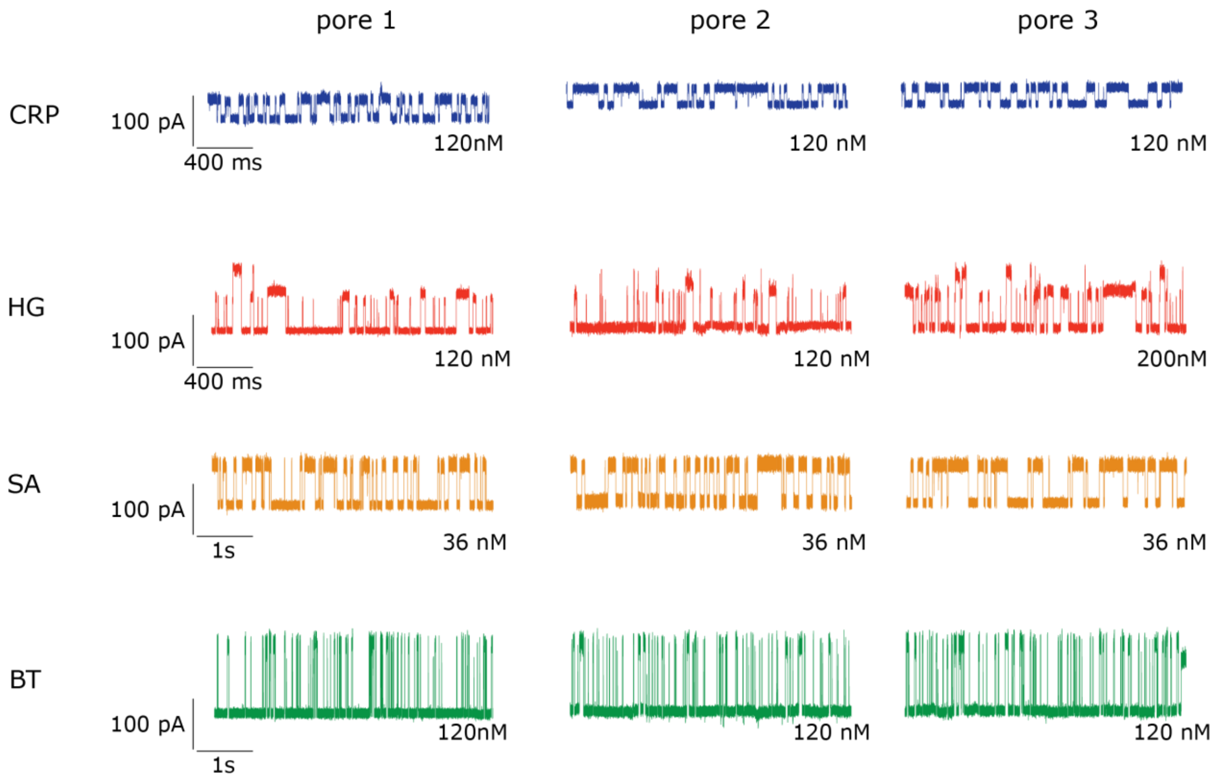

Figure S13: Representative traces of protein capture by YaxA$_{\Delta40}$B^{80} in triplicate. The final concentration of each protein is indicated below the trace; proteins were added to *cis* chamber and recorded at -75 mV. Experiments were performed in 150 mM NaCl, 15 mM Tris-HCl pH 7.5. Data was collected at 50 kHz sampling rate and 10 kHz Bessel filter. Data was filtered for analysis with 5 kHz low-pass Gaussian filter for visualization.

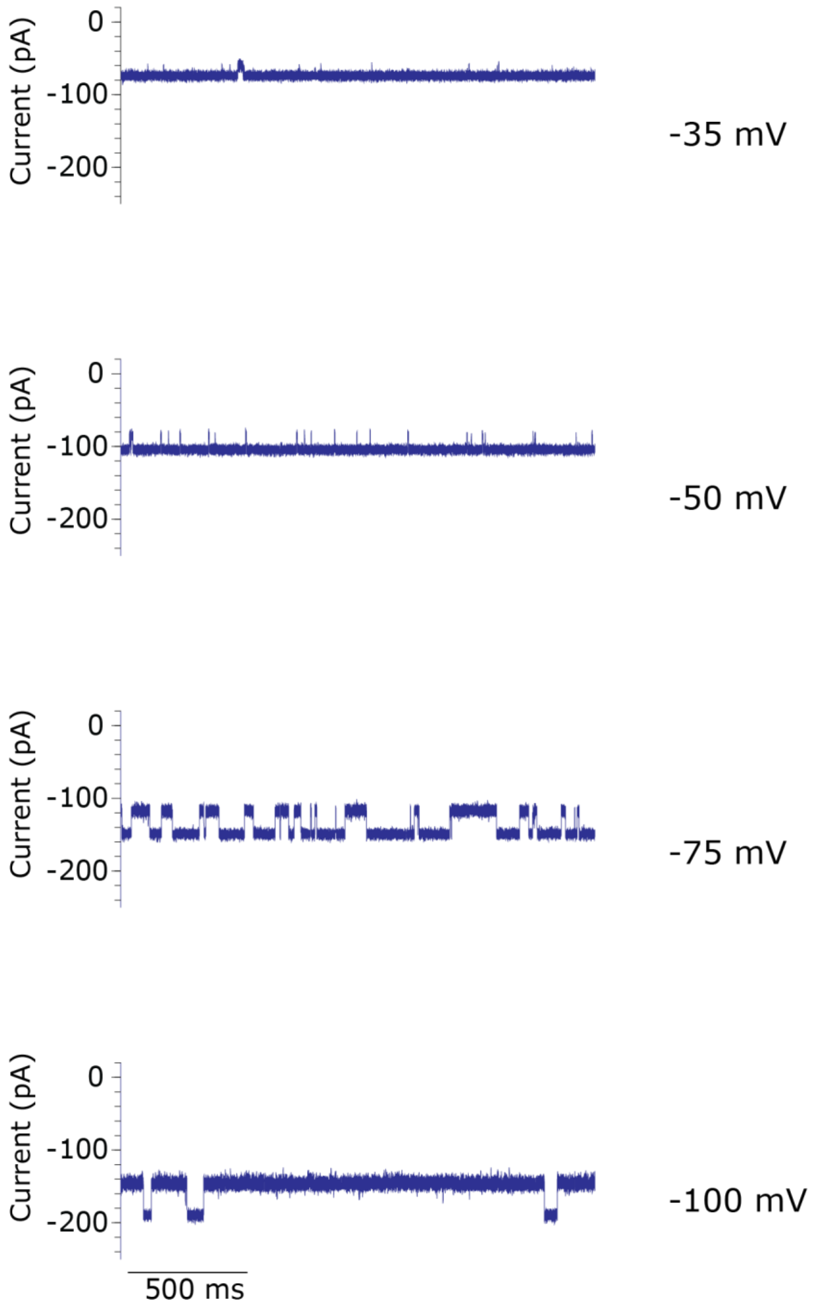

Figure S14: CRP capture in YaxA$_{\Delta 40}$B^{80} at increasing voltage. The dwell time of CRP increases with voltage. See Fig S19 for quantification of capture and release frequency. 80 nM CRP was added to *cis*. Experiments were performed in 150 mM NaCl, 15 mM Tris-HCl pH 7.5. Sampling frequency was set at 50 kHz, with 10 kHz Bessel filter. Traces were additionally filtered with 5 kHz low-pass Gaussian filter for visualization.

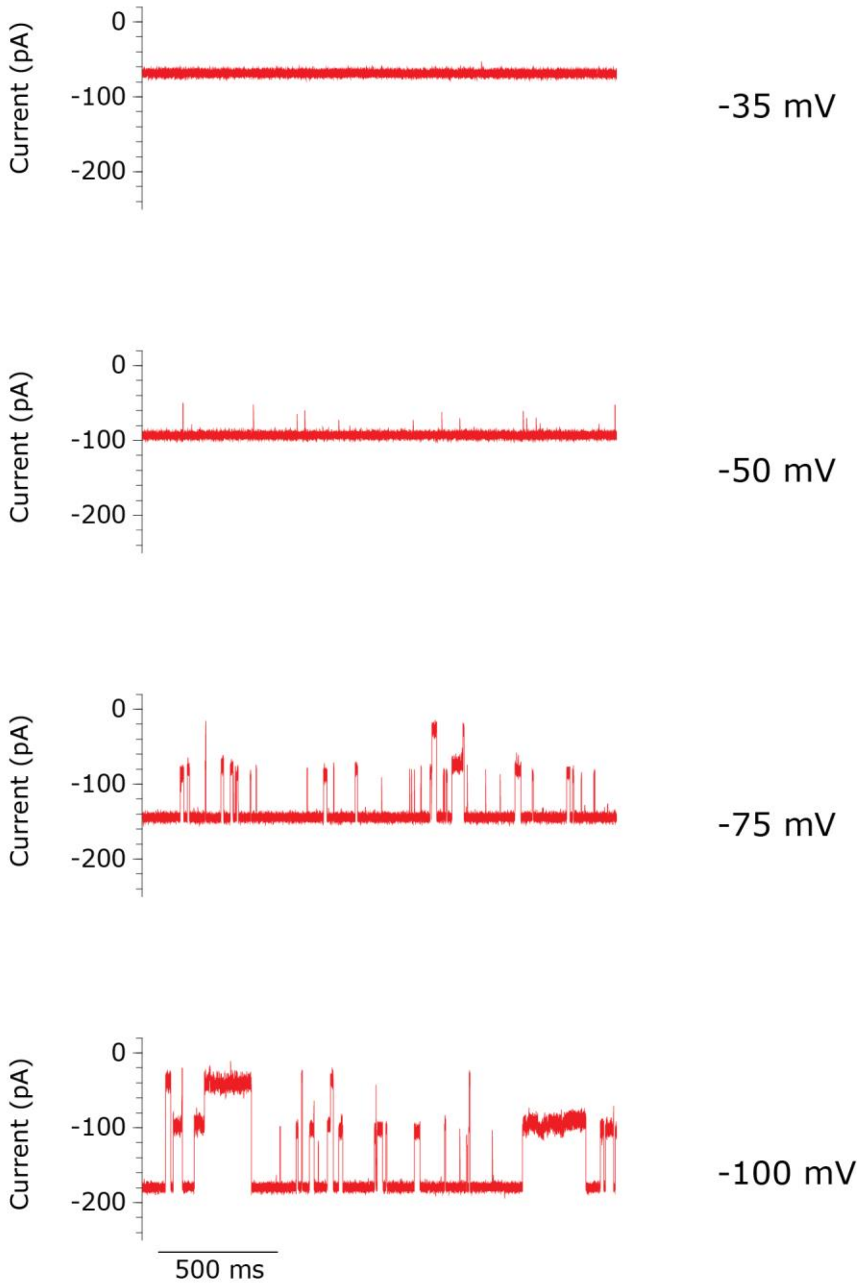

Figure S15: HG capture in YaxA$_{\Delta40}$B^{80} at increasing voltage. The dwell time of HG increases with voltage. See Fig S19 for quantification of capture and release frequency. 80 nM HG was added to *cis*. Experiments were performed in 150 mM NaCl, 15 mM Tris-HCl pH 7.5. Sampling frequency was set at 50 kHz, with 10 kHz Bessel filter. Traces were additionally filtered with 5 kHz low-pass Gaussian filter for visualization.

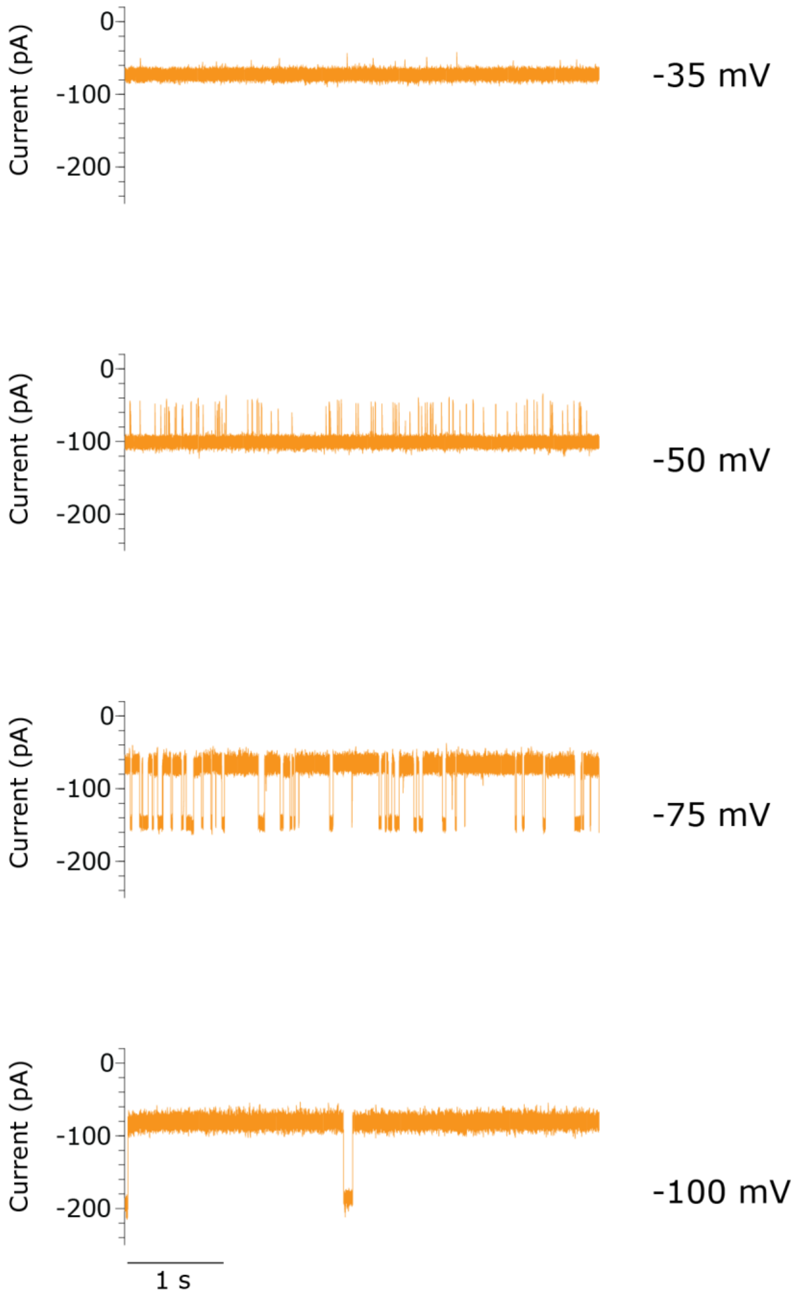

Figure S16: SA capture in YaxA$_{\Delta 40}$B^{80} at increasing voltage. The dwell time of SA increases with voltage. See Fig S19 for quantification of capture and release frequency. 80 nM SA was added to *cis*. Experiments were performed in 150 mM NaCl, 15 mM Tris-HCl pH 7.5. Sampling frequency was set at 50 kHz, with 10 kHz Bessel filter. Traces were additionally filtered with 5 kHz low-pass Gaussian filter for visualization.

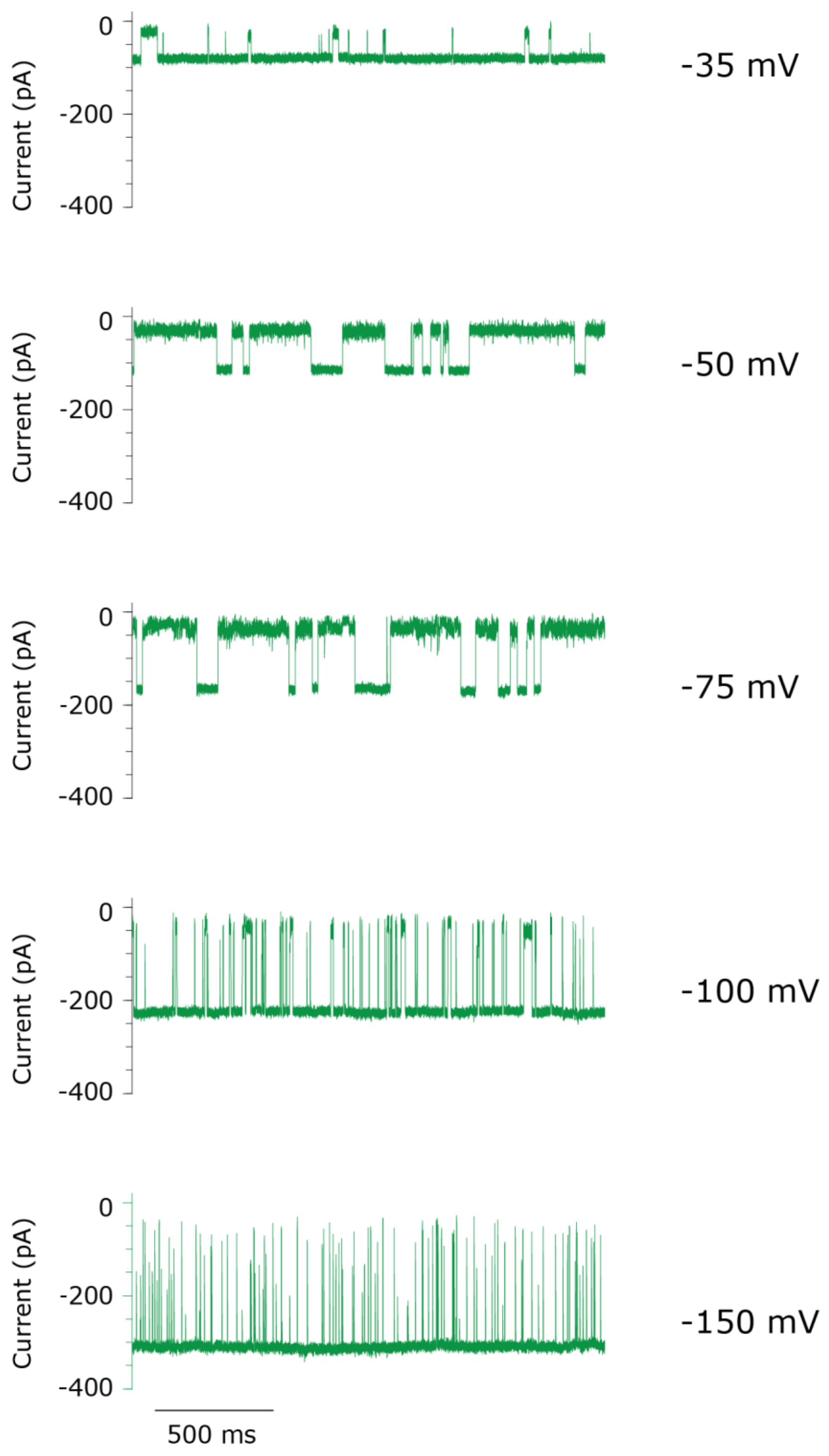

Figure S17: BT capture in YaxA$_{\Delta 40}$B^{80} at increasing voltage. The dwell time of BT initially increases (-35 to -50 mV) and then decreases (-50 mV to -150 mV), suggesting translocation of BT at higher voltages. See Fig S19 for quantification of capture and release frequency. 200 nM BT was added to *cis*. Experiments were performed in 150 mM NaCl, 15 mM Tris-HCl pH 7.5. Sampling frequency was set at 50 kHz, with 10 kHz Bessel filter. Traces were additionally filtered with 5 kHz low-pass Gaussian filter for visualization.

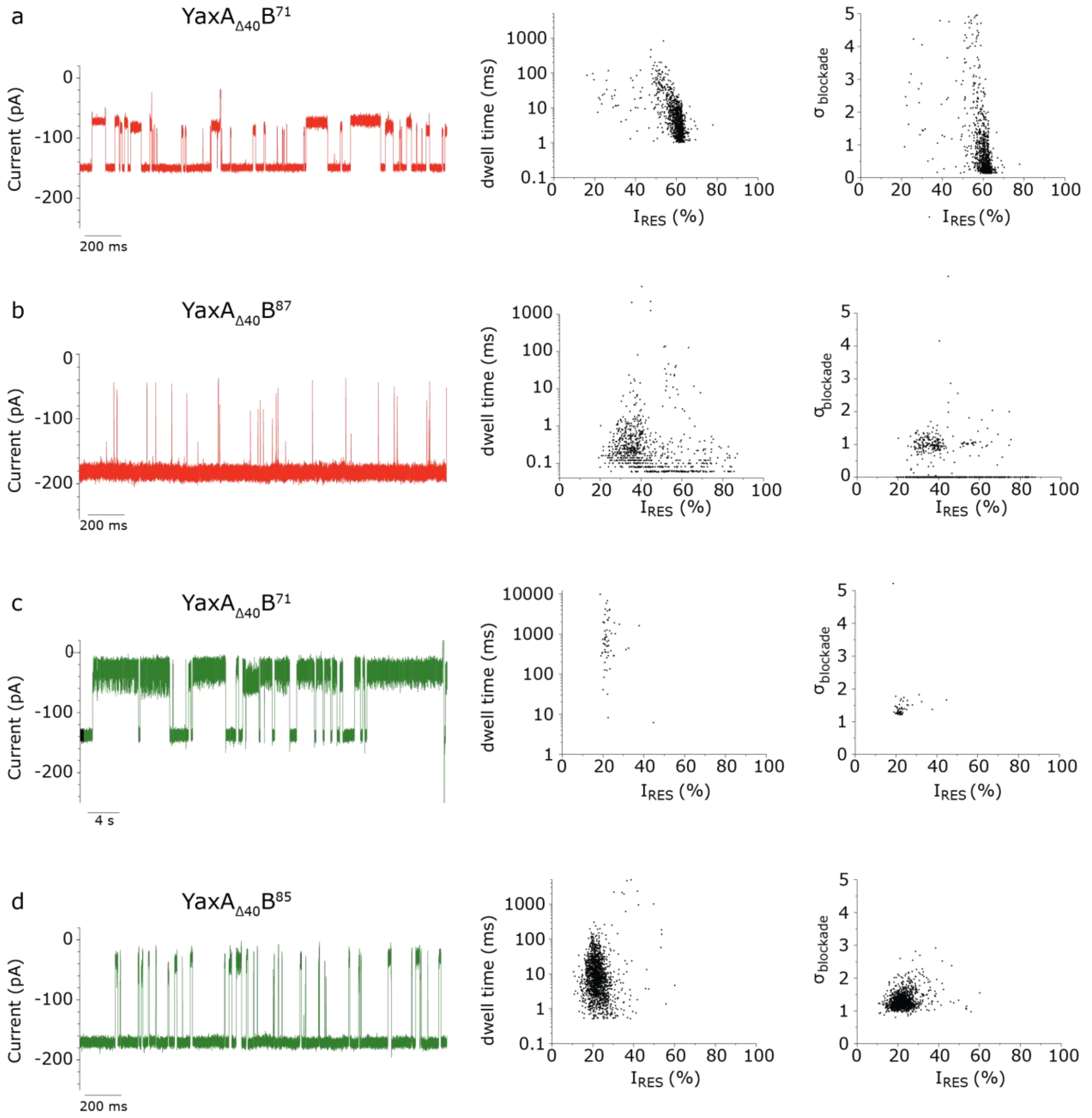

Figure S18: HG and BT trapping in YaxA$_{\Delta40}$B with different conductance. **a)** 120 nM HG added to *cis* of a pore with YaxA$_{\Delta40}$B^{71} at -35 mV recorded for ~1 min, and **b)** 50 nM HG added to *cis* of a pore with YaxA$_{\Delta40}$B^{87} at -35 mV, recorded for ~1 min. **c)** 20 nM BT added to *cis* of pore with YaxA$_{\Delta40}$B^{71}at -35 mV, recorded for ~2 min, and **d)** 120 nM BT added to *cis* of a pore with YaxA$_{\Delta40}$B^{87} at -35 mV, recorded for ~2 min. Measurements were performed in 150 mM NaCl, 15 mM Tris-HCl pH 7.5 at -75 mV, in triplicate. Data was collected at 50 kHz sampling rate and 10 kHz Bessel filter. Data was filtered for analysis with 5 kHz low-pass Gaussian filter for visualization.

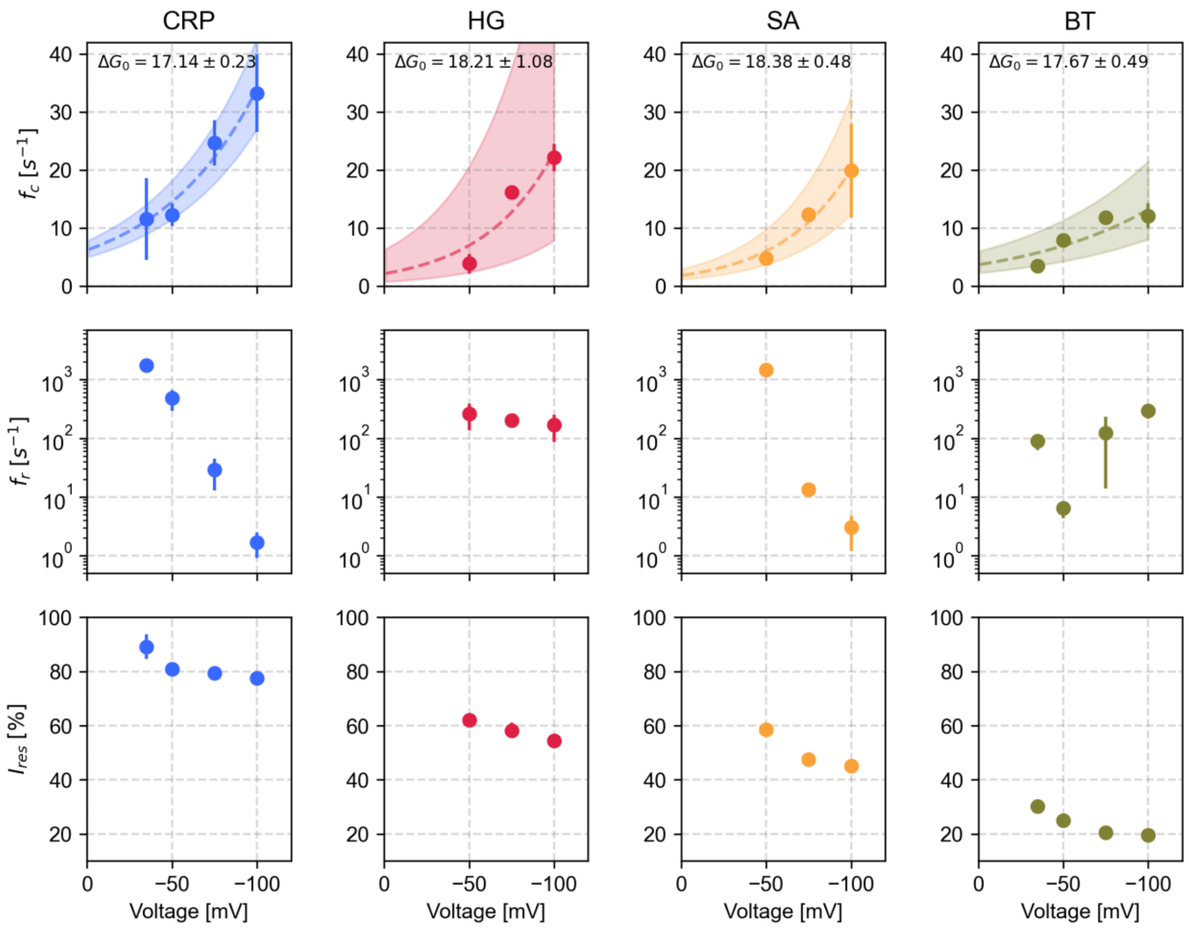

Figure S19. Voltage-dependent capture of folded proteins in YaxA$_{\Delta40}$B^{80}. Capture frequency (f_c, **upper panels**), release frequency (f_r, **middle panels**), and I_{RES} (**lower panels**) of CPR, SA, HG, and BT in YaxA$_{\Delta40}$B^{80} as function of voltage. Proteins were added to *cis* at 80 nM. Symbols represent the average of the measurements with error bars being the standard deviation of three independent experiments (N=3). The shaded area represents the 95% confidence bands of the nonlinear regression using the bootstrap method. Measurements were performed in 150 mM NaCl, 15 mM Tris-HCl pH 7.5, at 50 kHz sampling rate and 10 kHz Bessel filter. $\Delta G_0[k_BT]$ is the entrance free-energy barrier of each protein in the nanopore, at equilibrium, fitted by using the analytical formulas derived by ref.[6], see **Supplementary Note**. The analysis hypothesizes that the entrance free energy barriers dominate the capture frequency for the four proteins.

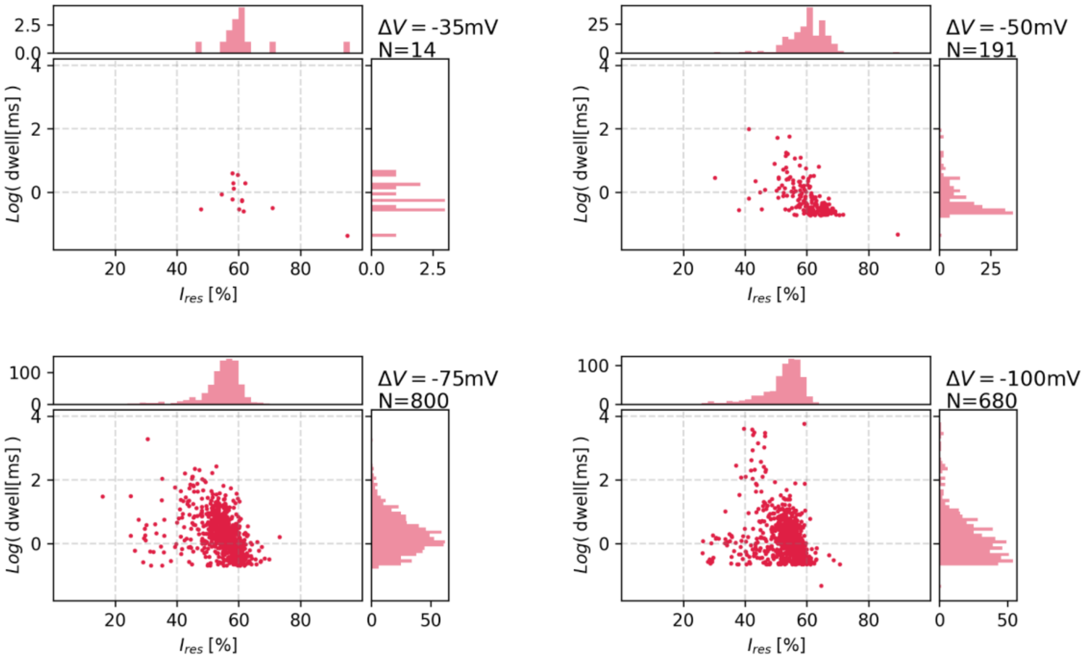

Figure S20: Hemoglobin capture at different voltages. The scatter plots of the detected events for HG at different voltages showed that increasing the voltages increases the relative number of longer detected events having a slightly lower I_{RES}. The data suggest the presence of a stable position deeper inside the nanopore for the HG molecule that can be easier reached at larger voltages. 80 nM HG was added to *cis*, and recorded for 1 min at the given voltage. Measurements were performed in 150 mM NaCl, 15 mM Tris-HCl pH 7.5, at 50 kHz sampling rate and 10 kHz Bessel filter.

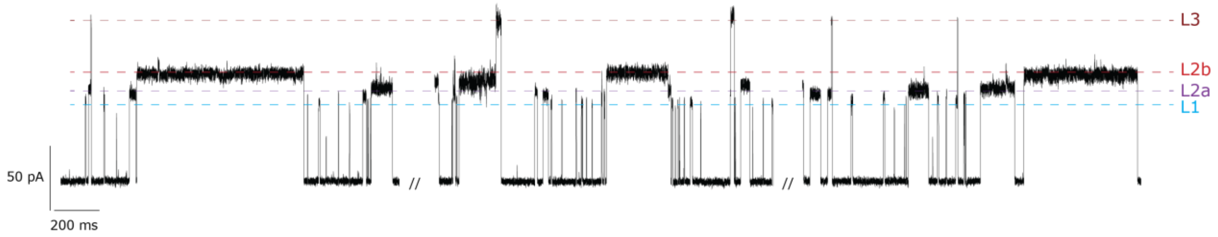

Figure S21: Haemoglobin sublevel analysis as detected by YaxA$_{\Delta40}$B^{80}. HG showed multiple non-uniform blockades that could be annotated as 3 levels (L1, L2, L3). Among the L2 events, we could discriminate a subgroup of events with shorter (L2a) and longer (L2b) dwell time. Representative traces were recorded with 120 nM HG added to *cis*, at -75 mV applied potential. Experiments were performed in 150 mM NaCl, 15 mM Tris-HCl pH 7.5, recorded at 50 kHz sampling frequency and 10 kHz Bessel filter. Data was additionally filtered with 2 kHz low-pass Gaussian filter for analysis and visualization.

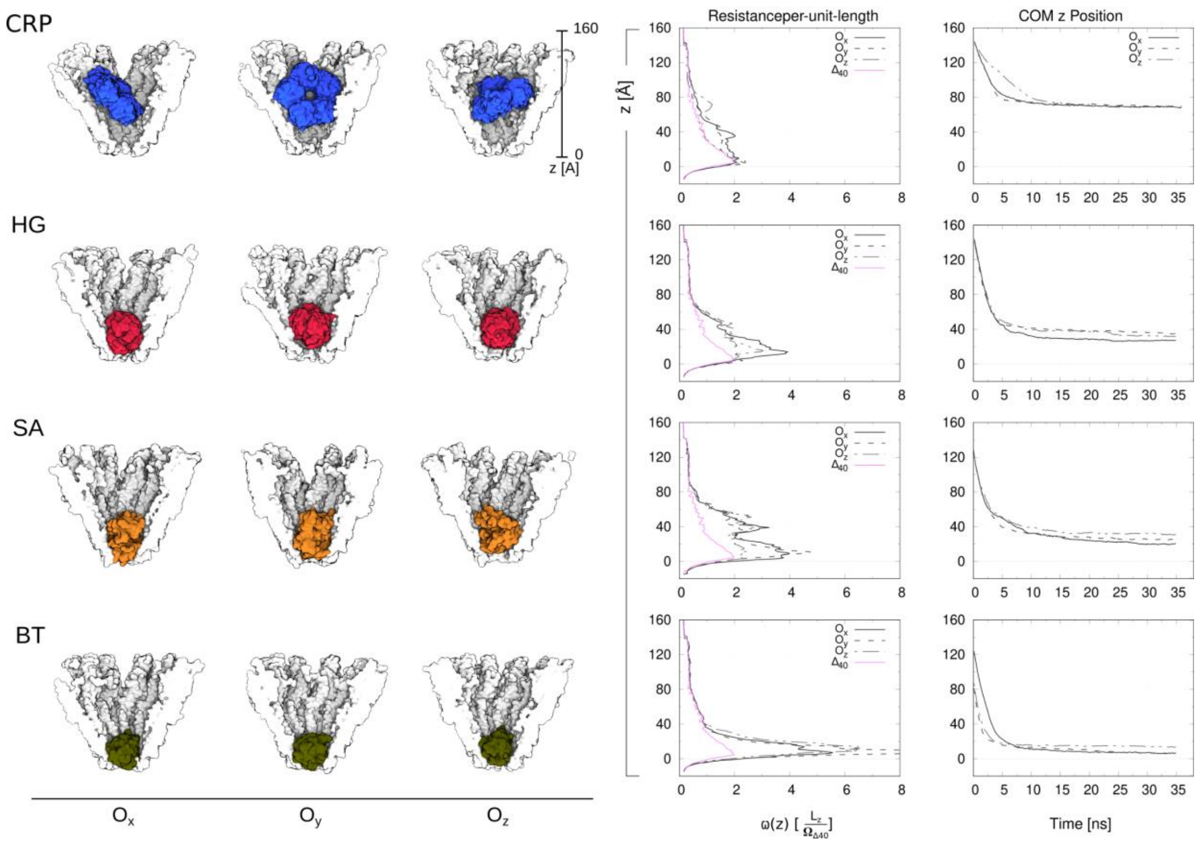

Figure S22. Steered MD (SMD) simulations and hindrance estimation of the CRP, SA, HG and BT proteins inside the decameric YaxA$_{\Delta 40}$B. Pictures display the final configuration (last frame) of each SMD simulation, with a F = 10.0 kcal/(mol Å) = 690 pN total force applied over all the protein (colored) atoms. Electrolyte and membrane not shown. For each protein three SMD are performed, starting from different initial configurations: Ox, Oy, Oz. The three initial configurations are prepared by aligning the main inertia axis of the protein along the x,y,z-axis, respectively. Plots (right columns) report the position of the center of mass of the proteins during the simulation and the resistance-per-unit-length profiles, along the pore axis z, computed over the last 8 ns of each simulation. The resistance-per-unit-length $\omega(z)$ is normalized by the average resistance-per-unit-length of the YaxA$_{\Delta 40}$B system, $\omega_{\Delta 40} = \frac{\Omega_{\Delta 40}}{L_z} = 26 \times 10^{12}\ \Omega/nm$, with $\Omega_{\Delta 40}$ total resistance and $L_z = 15\ nm$ length of the pore.

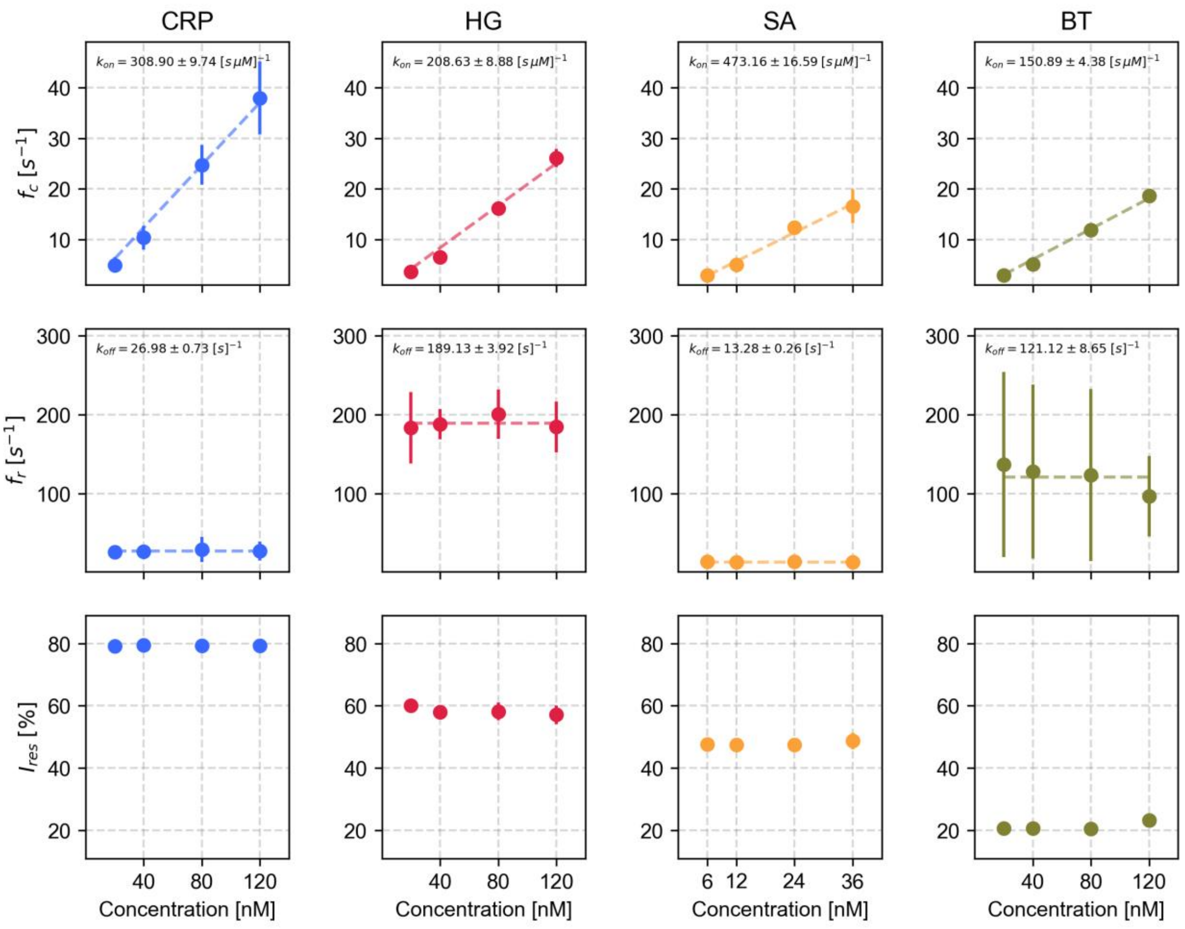

Figure S23. Concentration-dependent capture of folded proteins in YaxA$_{\Delta 40}$B^{80}. Capture frequency f_c (**upper panels**), release frequency f_r (**middle panels**), and I_{RES} (**lower panels**) of CRP, SA, HG and BT in YaxA$_{\Delta 40}$B^{80} at increasing concentration, measured at -75 mV. Proteins were added to *cis* at 20-120 nM. Symbols represent the average of the measurements with error bars being the standard error of three independent experiments (N=3). Measurements were performed in 150 mM NaCl, 15 mM Tris-HCl pH 7.5, at 50 kHz sampling rate and 10 kHz Bessel filter. Dashed lines in **top panels** represent the least squares fit of the linear function $f_c = k_{on}[POI]$ with [POI] the concentration of the analytes and k_{on} the slope of the curve. The resulting fitted k_{on} are reported in each panel, with the error being the mean squared regression error of the fit. Dashed lines in the **middle panels** represent the least squares fit of the function $f_r = k_{off}$, since the data suggest that the release frequency does not depend on the concentration of the analytes. The resulting fitted k_{off} are reported in each panel, with the error being the mean squared regression error of the fit (roughly equivalent to the standard error of the mean).

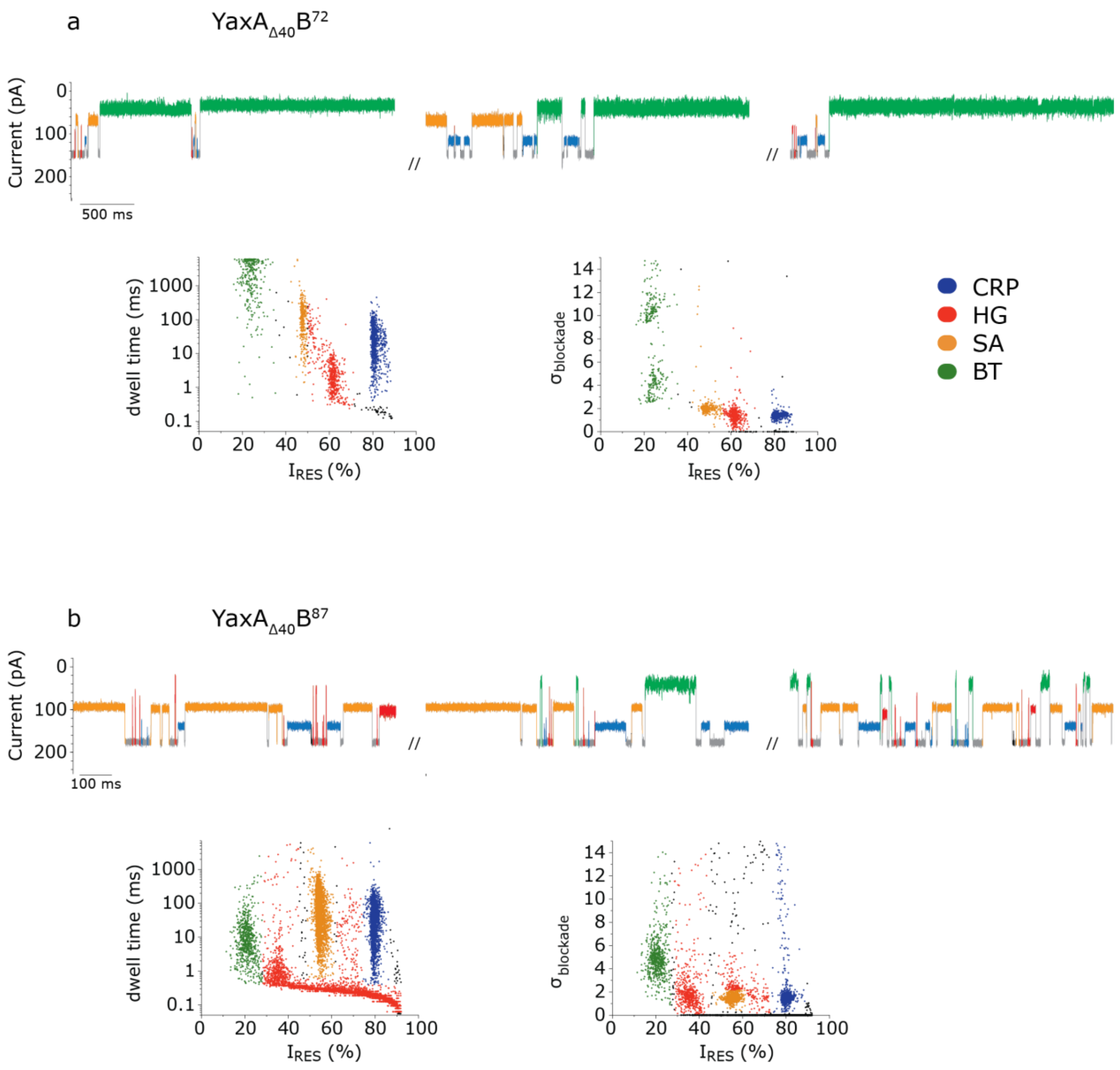

Figure S24: Resolution of mixed sample with YaxA$_{\Delta40}$B pores with different conductance. Electrophysiology trace and corresponding dwell time vs I_{RES} and $\sigma_{blockade\ vs\ IRES}$ scatterplots of **a)** 150 nM HG, 50 nM BT, 50 nM CRP, 50 nM SA added to *cis* of a pore with -72 pA at -35 mV; and **b)** 50 nM HG, 50 nM BT, 50 nM CRP, 20 nM SA added to *cis* of pore with -87 pA at -35 mV. Smaller YaxA$_{\Delta40}$B pore gives longer BT and HG blockades (see also **Figure S18**) and therefore each of the four proteins is separated in the scatterplots. Notably, the larger YaxA$_{\Delta40}$B pore lowered the dwell time for HG dramatically, and L3 blockades became more prominent. The larger pore also has a partial overlap of L2 HG blockades with SA in the dwell time vs I_{RES} scatterplot, which could be separated in the $\sigma_{blockade\ vs\ IRES}$ scatterplot. Overall, the larger YaxA$_{\Delta40}$B^{87} pore thus gave lower resolution in separating the four proteins. The resolution displayed by YaxA$_{\Delta40}$B^{80} pores fall in between these examples, we showed a YaxA$_{\Delta40}$B^{75} pore in **Fig. 3c** in main text with optimal resolution. Experiments were performed in 150 mM NaCl, 15 mM Tris-HCl pH 7.5, at -75 mV, in duplicate. (a) was recorded for ~22 min, n=2045 datapoints. (b) was recorded for ~11 min, n=8868 datapoints. Data was recorded at 50 kHz sampling rate, and 10 kHz Bessel filter. Traces were additionally filtered with 5 kHz low-pass Gaussian filter for visualization.

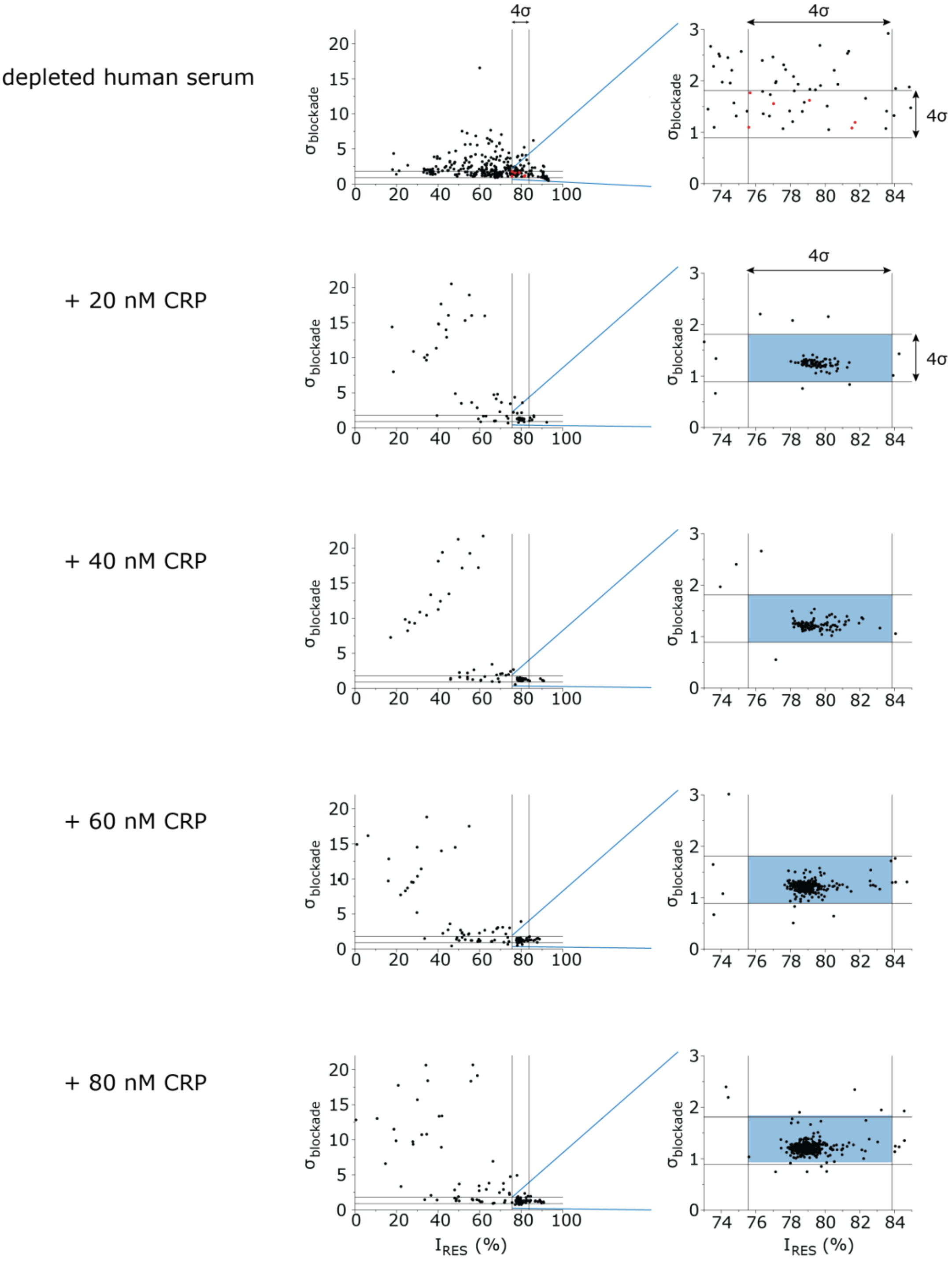

Figure S25: $\sigma_{blockade}$ vs I_{RES} scatterplot of CRP events in the presence of proteins from depleted human serum. The figure is an extension of Fig. 4 in main text. **Top panel**: depleted serum proteins detected by $YaxA_{\Delta 40}B^{80}$ during 10 min recording. The boundaries indicate potential CRP events (see Methods). Red datapoints are events that correspond to I_{RES}, $\sigma_{blockade}$, and dwell time characteristics of CRP. **Lower panels**: CRP titrated on top of proteins from depleted serum, detected by $YaxA_{\Delta 40}B^{80}$. Traces were recorded for 2 min. Experiments were performed in 150 mM NaCl, 15 mM Tris-HCl pH 7.5, with 50 kHz sampling rate and 10 kHz low-pass Bessel filter. For analysis, data was filtered with additional 5 kHz low-pass Gaussian filter.

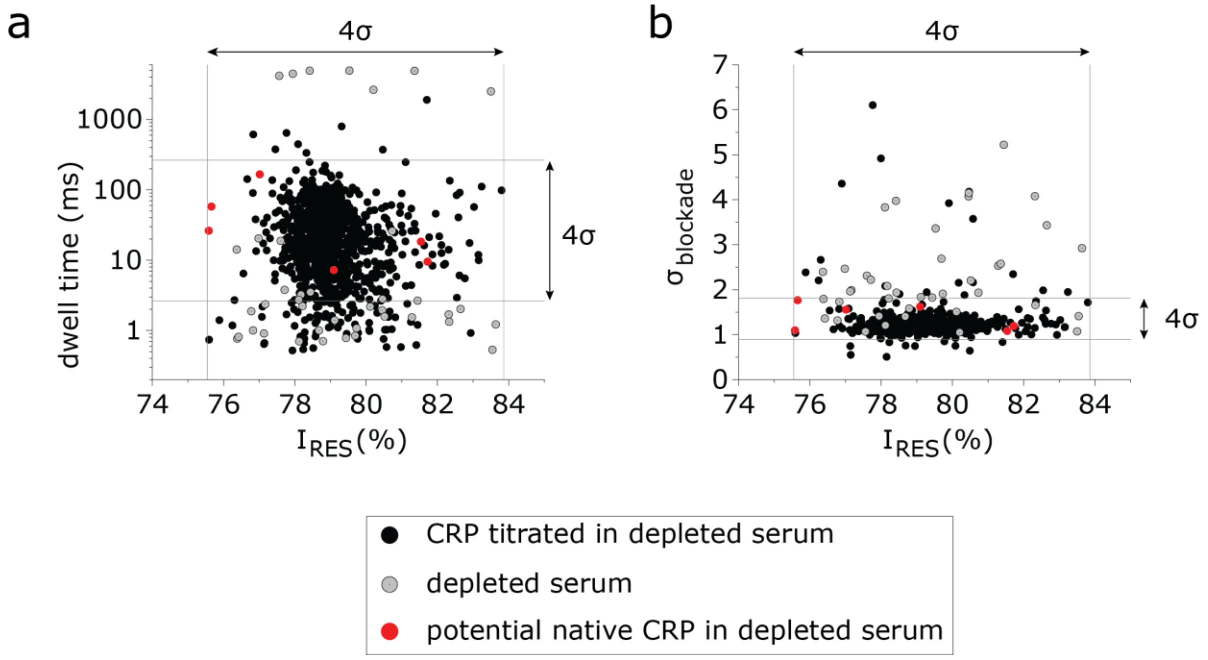

Figure S26: Interpreting potential CRP events in depleted serum. All annotated CRP events from CRP titrations in the presence of proteins in depleted serum are compiled in black (n=1040 events, N=2 pores). As a reference, the CRP events annotated from proteins in depleted serum are in grey (n=46, N=1 pore; see Methods). **a)** Dwell time vs I_{RES} scatterplot with 2σ-boundaries include 16 depleted serum events within both borders. **b)** $\sigma_{blockade}$ vs I_{RES} scatterplot with respective 2σ-boundaries include 21 depleted serum events. Six events are included in I_{RES}–, $\sigma_{blockade}$– and dwell time–boundaries: events #2, 9, 31, 32, 39, 46 (red, **Table S2**). Most events are excluded from either reference. Experiments were performed in 150 mM NaCl, 15 mM Tris-HCl pH 7.5, with 50 kHz sampling rate and 10 kHz low-pass Bessel filter. For analysis, data was filtered with additional 5 kHz low-pass Gaussian filter.

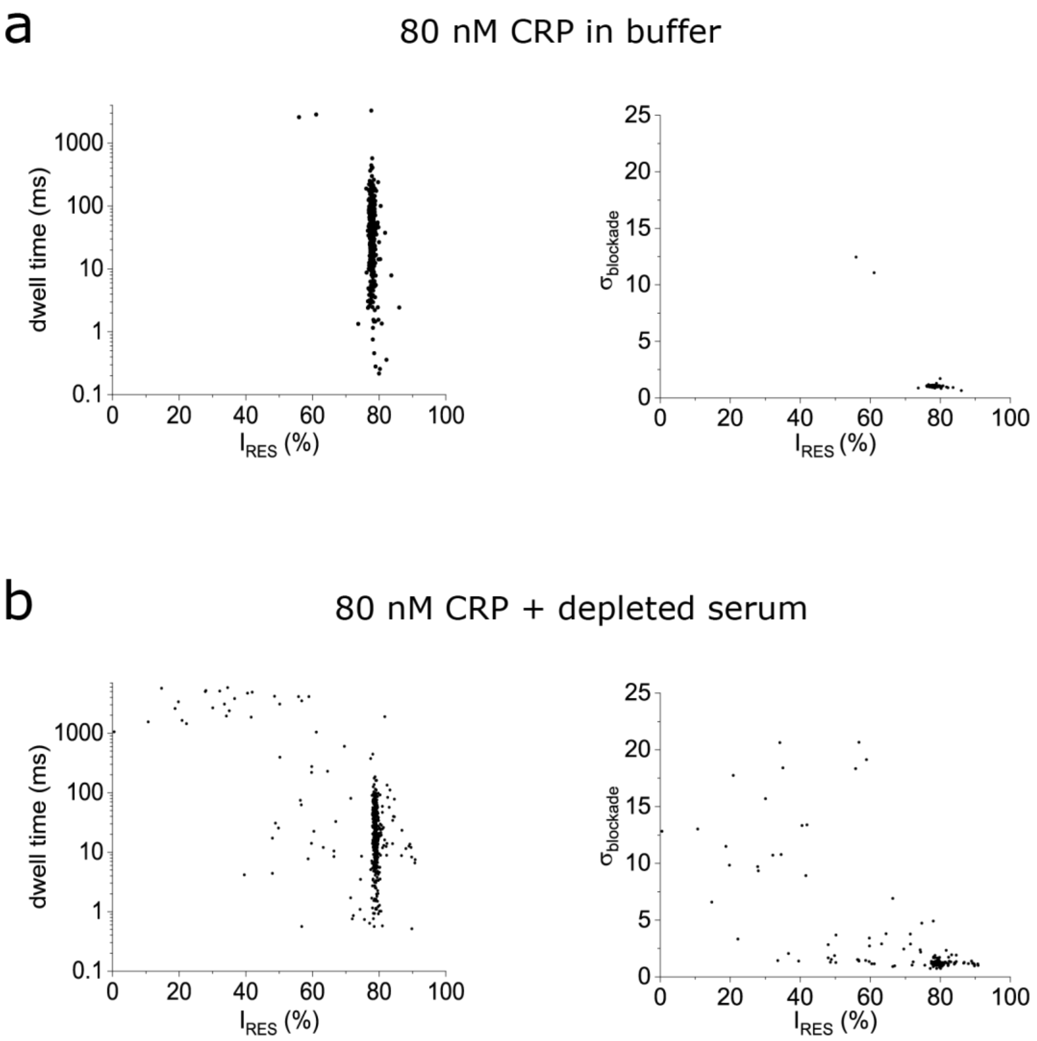

Figure S27: Comparison between CRP in buffer and CRP with proteins in depleted serum. a) 80 nM CRP in buffer. Dwell time vs I_{RES} (left) and $\sigma_{blockade}$ vs I_{RES} (right). **b)** 80nM CRP in the presence of protein from depleted serum. Dwell time vs I_{RES} (**left**) and $\sigma_{blockade}$ vs I_{RES} (**right**). Both conditions showed clusters with similar distribution around I_{RES} ~80%. Experiments were performed in 150 mM NaCl, 15 mM Tris-HCl pH 7.5, with 50 kHz sampling rate and 10 kHz low-pass Bessel filter. For analysis, data was filtered with additional 5 kHz low-pass Gaussian filter.

Table S1: **Biophysical values of the proteins: experimentally fitted vs. theoretical.** Entrance free-energy barrier (ΔG_0) and charge (q) are fitted parameters in **Figure S19** and **Figure S23**. Hydrodynamic radius (r_h) was fixed, and was computed from a sphere of volume equal to that of the expanded convex hull corrected by a shape factor, using the HullRad[7] software (version 9, October 2022). The sequence charge (q_{seq}) is the charge computed by the protein sequence, assigning the nominal charge -1 for Asp/Glu residues and +1 for Lys/Arg. The predicted charge (q_{pKa}) is the charge computed by using Propka[8] software, accounting for the 3D structure of the protein and the local pKa for each residue. The fitted charge (q_{fit}) is the charge fitted in **Fig. S19** using the analytical capture model described in the **Supplementary Note**. For SA and HG, the fitted and predicted values are in good agreement and close to zero for both cases. For BT and CRP, the fitted charge is lower than the predicted one. For CRP, there is a discrepancy between the nominal charge and reported experimental values. Despite the large number of negative charges present in its sequence, CRP acts as a neutral molecule in electrolyte solutions at pH ~ 7 (from ref.[9]). Also, in our capture experiments (**Fig. S19**), the protein acts essentially as an uncharged or positively charged particle: the capture rates increment at larger negative voltages (see reference system of **Fig. 3a**), and release rates exponentially decrease.

**protein**	**ΔG_0 = [k_BT]**	**r_h [Å]**	**q_{seq} [e]**	**q_{fit} [e]**	**q_{pKa} [e]**	**PDB code**
CRP	17.14 ± 0.23	43.7	-20	-0.6 ± 0.4	-15.6	1GNH
HG	18.21 ± 1.08	33.1	2	1.2 ± 1.8	0.2	1B86
SA	18.38 ± 0.48	31.6	0	1.4 ± 0.8	1.5	6J6J
BT	17.67 ± 0.49	30.4	6	-0.3 ± 0.9	3.8	1MKW

Table S2: Possible CRP events in depleted human serum. A potential CRP event was defined by I_{RES} ± 2σ, with corresponding normalized standard deviation of the event $σ_{blockade}$, and dwell time. Numbers in red: within either CRP-like boundary from **Figure S26**. Events also marked grey (#2, 9, 31, 32, 39, 46): within all CRP-like boundaries from **Table 1** in main text.

event #	I_{RES} (%)	$σ_{blockade}$	dwell time (ms)
1	78.12	1.21	0.7
2	81.73	1.19	9.52
3	78.17	2.08	3.22
4	80.12	1.51	2.18
5	76.83	1.73	1
6	77.17	1.99	2.36
7	83.5	1.07	2508.74
8	83.64	2.92	1.22
9	75.59	1.1	26.22
10	77.6	2.31	18.78
11	78.21	1.8	2.22
12	80.45	4.07	1.94
13	78.68	1.4	1
14	76.78	1.31	1.88
15	82.65	3.43	2.02
16	81.29	2.53	1.53
17	79.73	1.91	1.08
18	76.37	2.4	14.17
19	79.12	1.83	1.39
20	79.44	1.82	0.78
21	83.54	1.41	0.53
22	80.46	4.15	2.8
23	76.39	1.79	0.77
24	78.79	1.58	0.7
25	82.33	1.66	1.33
26	78.44	1.93	3.5

27	80.52	2.2	1.58
28	77.71	2.22	3.78
29	76.42	1.36	0.81
30	77.57	1.07	4173.91
31	79.1	1.62	7.22
32	81.54	1.08	18.31
33	82.32	4.08	1.69
34	80.21	1.05	2644.16
35	76.98	2.46	20.41
36	77.14	1.96	0.91
37	78.11	3.83	2.72
38	81.44	5.22	2.66
39	77.02	1.56	165.78
40	79.7	2.69	0.84
41	80.73	1.93	25.91
42	77.95	1.41	4471.84
43	81.36	2.58	4919.69
44	78.42	3.97	4936.06
45	79.53	3.36	4944.31
46	75.66	1.77	57.75

SUPPLEMENTARY NOTE

The exponential trends in the experimental capture frequency reported in **Figure S19** were fitted using the formulas derived by ref.[6] From a generalized Smoluchowski model, the authors derived different analytical expressions for the capture of a single molecule into a nanopore, under different regimes. For reader convenience, we report here the main assumptions and related equations used to fit the experimental data.

In general, the capture frequency f_c can be written as the sum of two contributions: one due to the particles approaching the pore (f_a) and one due to the particles actually entering the pore region (f_e). As a first step we show that the approach frequency contribution is negligible for our case. Indeed, the pure diffusive prediction by standard Smoluchowski formula $f_a = 2\pi\, D\, C_0\, r_e$ with $r_e = 7.5\, nm$ the pore entrance radius, C_0 the bulk concentration of the proteins and $D = k_B T/(\,6\pi\,\eta\, r_h\,)$ the diffusion coefficient, with r_h the hydrodynamic radius and the viscosity of water $\eta\ =\ 8.9\ \times\ 10^{-4}\, Pa\cdot s$, result to be two orders of magnitude larger than experimental ones. Hence, the capture frequency is dominated by the entrance barriers, $f_c \simeq f_e$. We then used the expression for the entrance-limited regimes, Eq. 29 of ref.[6], that reads

$$f_c\ \simeq f_e\ =\ 2\pi\, C_0 r_e^3\, (\frac{k_B T}{h})\, exp\, [\, 2\phi(r_e)\ -\ \frac{\Delta G_0}{k_B T}\,]\ ,$$

with $\phi(r_e) = -\frac{U(r_e)}{k_B T}$ dimensionless effective potential and $U(r_e)\ =\ -\frac{1}{2\pi r}\,[\,\frac{qI}{\sigma} + \frac{Q_f}{\mu}]$ effective potential, whose derivative with respect to r describe the advection and electrophoretic contribution due to the presence of an electroosmotic flow Q_f and an electrical field; $\mu\ =\ D\, k_B T$ is protein mobility, q its charge and I total electric current. In the above equations we neglected the dielectrophoretic contribution since the relative potential was much smaller than electrophoretic and electroosmotic contributions. Also, the electrophoretic contribution is difficult to evaluate, since it is not only related to the intrinsic protein charge (that can be derived by the protein sequence and structure[8]) but it highly depends on the high ionic strength of the electrolyte[10,11]. Since the experiments are conducted at 0.15M, the electrophoretic mobility would be largely decreased due to the counterion screening. Hence, as a first approximation, in the fits of **Figure S19**, we also fitted the charge (q), verifying a-posteriori that the net charge is close to zero for all the four proteins. The electric current and the electroosmosis were approximated to be proportional to the applied voltage ΔV: $I = G_c\, \Delta V$ and $Q_f\ =\ G_{eo}\, \Delta V$, with $G_c\ =\ \frac{100\, pA}{35\, mV}\ =\ 2.9\, nS$ and $G_{eo}\ =\ \frac{70\, molecules/ns}{125\, mV}\ =\ 16.7\, \mu m^3/(s\cdot V)\ =\ 16.7\, fL/(s\cdot V)$. G_{eo} is extracted from the MD simulations discussed in **Fig. 2**. The hydrodynamic radii and the fitted parameters are reported in **Table S1**.

References

(1) Huang, G.; Willems, K.; Bartelds, M.; Van Dorpe, P.; Soskine, M.; Maglia, G. Electro-Osmotic Vortices Promote the Capture of Folded Proteins by Plyab Nanopores. *Nano Lett.* **2020**, *20* (5), 3819–3827. https://doi.org/10.1021/acs.nanolett.0c00877.

(2) Narayanan Eswar; David Eramian; Ben Webb; Min-Yi Shen; Andrej Sali. Protein Structure Modeling with MODELLER. *Methods Mol. Biol.* **2008**, *426*, 145–159. https://doi.org/10.1007/978-1-60327-058-8_8.

(3) Humphrey, W.; Dalke, A.; Schulten, K. VMD: Visual Molecular Dynamics. *J. Mol. Graph.* **1996**, *14* (1), 33–38. https://doi.org/10.1016/0263-7855(96)00018-5.

(4) Bhattacharya, S.; Muzard, J.; Payet, L.; Mathé, J.; Bockelmann, U.; Aksimentiev, A.; Viasnoff, V. Rectification of the Current in α-Hemolysin Pore Depends on the Cation Type: The Alkali Series Probed by Molecular Dynamics Simulations and Experiments. *J. Phys. Chem. C* **2011**, *115* (10), 4255–4264. https://doi.org/10.1021/jp111441p.

(5) Di Muccio, G.; Rossini, A. E.; Di Marino, D.; Zollo, G.; Chinappi, M. Insights into Protein Sequencing with an α-Hemolysin Nanopore by Atomistic Simulations. *Sci. Rep.* **2019**, *9* (6440), 1–8. https://doi.org/10.1038/s41598-019-42867-7.

(6) Chinappi, M.; Yamaji, M.; Kawano, R.; Cecconi, F. Analytical Model for Particle Capture in Nanopores Elucidates Competition among Electrophoresis, Electroosmosis, and Dielectrophoresis. *ACS Nano* **2020**, *14* (11), 15816–15828. https://doi.org/10.1021/acsnano.0c06981.

(7) Fleming, P. J.; Fleming, K. G. HullRad: Fast Calculations of Folded and Disordered Protein and Nucleic Acid Hydrodynamic Properties. *Biophys. J.* **2018**, *114* (4), 856–869. https://doi.org/10.1016/j.bpj.2018.01.002.

(8) Olsson, M. H. M.; SØndergaard, C. R.; Rostkowski, M.; Jensen, J. H. PROPKA3: Consistent Treatment of Internal and Surface Residues in Empirical PKa Predictions. *J. Chem. Theory Comput.* **2011**, *7* (2), 525–537. https://doi.org/10.1021/ct100578z.

(9) Tsujimoto, M.; Inoue, K.; Nojima, S. *Purification and Characterization of Human Serum C-Reactive Protein 3*; 1983; Vol. 94. https://academic.oup.com/jb/article/94/5/1367/782521.

(10) Stellwagen, E.; Stellwagen, N. C. Electrophoretic Mobility of DNA in Solutions of High Ionic Strength. *Biophys. J.* **2020**, *118* (11), 2783–2789. https://doi.org/10.1016/j.bpj.2020.02.034.

(11) Alberty, R. A. A Study of the Variation of the Average Isoelectric Points of Several Plasma Proteins with Ionic Strength. *J. Phys. Chem.* **1949**, *53* (1), 114–126. https://doi.org/10.1021/j150466a009.
